# Small endohedral metallofullerenes: exploration of the structure and growth mechanism in the Ti@C_2*n*_ (2*n* = 26–50) family†
†Electronic supplementary information (ESI) available: additional figures of energy profiles, detailed information about the Car–Parrinello simulations (including two movies) and optimized geometries for the most representative structures. See DOI: 10.1039/c4sc02268h



**DOI:** 10.1039/c4sc02268h

**Published:** 2014-09-12

**Authors:** Marc Mulet-Gas, Laura Abella, Paul W. Dunk, Antonio Rodríguez-Fortea, Harold W. Kroto, Josep M. Poblet

**Affiliations:** a Departament de Química Física i Inorgànica , Universitat Rovira i Virgili , Marcellí Domingo s/n , 43007 Tarragona , Spain . Email: antonio.rodriguezf@urv.cat ; Email: josepmaria.poblet@urv.cat; b Department of Chemistry and Biochemistry , Florida State University , Tallahassee , Florida 32306 , USA . Email: kroto@chem.fsu.edu

## Abstract

The formation of the smallest fullerene, C_28_, was recently reported using gas phase experiments combined with high-resolution FT-ICR mass spectrometry. An internally located group IV metal stabilizes the highly strained non-IPR C_28_ cage by charge transfer (IPR = isolated pentagon rule). Ti@C_44_ also appeared as a prominent peak in the mass spectra, and U@C_28_ was demonstrated to form by a bottom-up growth mechanism. We report here a computational analysis using standard DFT calculations and Car–Parrinello MD simulations for the family of the titled compounds, aiming to identify the optimal cage for each endohedral fullerene and to unravel key aspects of the intriguing growth mechanisms of fullerenes. We show that all the optimal isomers from C_26_ to C_50_ are linked by a simple C_2_ insertion, with the exception of a few carbon cages that require an additional C_2_ rearrangement. The ingestion of a C_2_ unit is always an exergonic/exothermic process that can occur through a rather simple mechanism, with the most energetically demanding step corresponding to the closure of the carbon cage. The large formation abundance observed in mass spectra for Ti@C_28_ and Ti@C_44_ can be explained by the special electronic properties of these cages and their higher relative stabilities with respect to C_2_ reactivity. We further verify that extrusion of C atoms from an already closed fullerene is much more energetically demanding than forming the fullerene by a bottom-up mechanism. Independent of the formation mechanism, the present investigations strongly support that, among all the possible isomers, the most stable, smaller non-IPR carbon cages are formed, a conclusion that is also valid for medium and large cages.

## Abstract 2

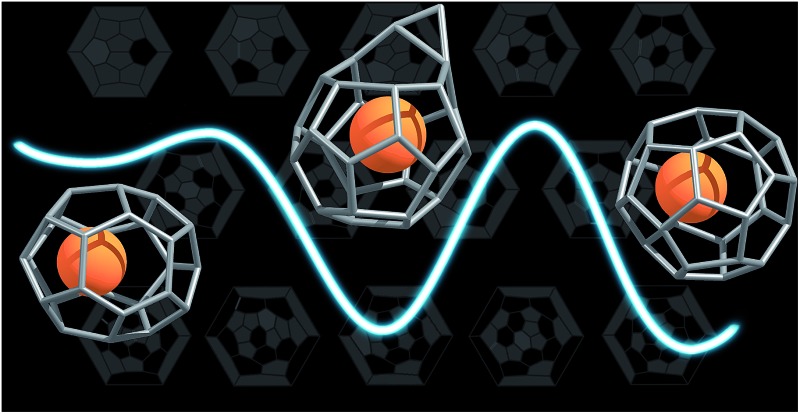
Analysis of the structure and the bottom-up growth mechanism in the family of small endohedral metallofullerenes Ti@C_2*n*_ (2*n* = 26–50).

## 1. Introduction

Since the discovery of C_60_ in 1985 by applying laser technology for the evaporation of graphite,^1^ tremendous advances have been made in the science of fullerenes and related compounds. Although soon after the discovery of C_60_ the first endohedral fullerene La@C_60_ was detected,^2^ the characterization in 1999 of Sc_3_N inside a cage with 80 carbons represented a qualitative change in the science of this new family of compounds; Sc_3_N@C_80_(*I*_h_) was found as the third most abundant fullerene after C_60_ and C_70_.^3^ During the last fifteen years, the number of known endohedral fullerenes has been constantly growing and carbon cages are able to encapsulate trimetallic nitrides, metallic oxides, metallic sulfides, metallic carbides, as well as up to three individual metal atoms.^4–9^ Electron transfer from the internal cluster to the carbon cage, which preferentially concentrates on the pentagonal rings, together with the maximum separation of the twelve pentagons and the metal–cage interaction in non-IPR cages, help us to understand and predict the observed endohedral compounds.^10,11^ It is worth mentioning that despite the complexity of the presumed formation mechanisms, in general the observed and predicted cages always coincide, and the most favorable thermodynamic isomer is usually observed.

In all synthetic procedures of carbon nanoforms, carbon needs to be exposed to very high temperatures,^12–14^ and the condensation process of carbon units seems to play a crucial role in the self-assembly of the fullerene cages.^15–17^ Several models have been suggested to explain the formation of fullerenes, among them the “party line”,^18^ the “pentagon road”,^19^ the “fullerene road”,^15^ the “ring-stacking”,^15,20^ and the “ring fusion spiral zipper” mechanisms.^21,22^ All of these models are based on the same concept, *i.e.* growing up from intermediate structures and additions of small C_*x*_ units in a thermodynamic equilibrium. Systematic QM/MD simulations of cooling carbon vapour, performed by Irle, Morokuma and co-workers, lead to the general conclusion that giant fullerene cages are spontaneously formed and that their sizes decrease *via* the so-called “Shrinking Hot Giant” (SHG) road.^23^ The same authors also remarked that fullerenes with a number of atoms lower than 100 tend to grow, as observed in molecular dynamics simulations at constant temperature and constant carbon density.^24^ Kinetic models provided an interpretation of the driving force of the shrinking process and the prevalence of C_60_ and C_70_ over other fullerenes.^25,26^ Recently, some of us shed new light on the fundamental processes that govern the self-assembly of carbon networks, showing that fullerenes self-assemble through a Closed Network Growth (CNG) mechanism by incorporation of C_2_ and atomic carbon.^27^ C_60_ exposed to the characteristic high synthesis temperature achieved by laser ablation, results predominantly in C_60_ with only very minor amounts of C_58_, C_56_, or C_54_, and a very low abundance of clusters larger than 60 carbon atoms. In contrast, in the presence of carbon vapour under the same conditions, remarkable amounts of C_60+2*n*_ species are observed, with C_70_ being formed at a higher relative abundance because of the IPR nature of the isomer.^27^

Less is known about the formation of endohedral fullerenes. However, it is more or less assumed that their formation mechanisms should not be rather different from those of empty cages. Indeed, Dunk *et al.* experimentally demonstrated that U@C_28_ is formed in a bottom-up growth mode and that it is the precursor for larger uranofullerenes.^28^ The studies conducted were also able to demonstrate that the tetravalent Ti^4+^ cation is encapsulated by a series of non-IPR carbon cages between C_28_ and C_50_, in which Ti@C_28_ and Ti@C_44_ appear as the most prominent peaks in the mass spectra.^28^

Understanding how these small M@C_2*n*_ form is essential to uncovering the mysteries of endohedral metallofullerene (EMF) formation. In this work, by use of comprehensive quantum chemical investigations, we study their formation using titanium encapsulated fullerene cages as a model. In particular, we show that once a Ti@C_2*n*_ carbon cage has already been formed, the addition of an extra C_2_ to the fullerene is strongly exergonic (between 130 and 170 kcal mol^–1^), with relatively small energy barriers taking into account the temperatures involved in the reaction. It is worth mentioning that all of the most favourable endohedral isomers are formally linked by a simple C_2_ addition, or by a C_2_ addition followed by a Stone–Wales (SW) transformation, and that the most abundant endohedral fullerenes correspond to cages that present a particular stability after a four electron transfer from the metal to the carbon cages. All these features seem only compatible with a relatively simple mechanism such as the one proposed from carbon vapour experiments by Kroto and co-workers.^27^ Very recently Balch, Olmstead, Dorn and co-workers interpreted the transformation of larger EMFs (C_84_ → C_80_) *via* the loss of C_2_ units and SW transformation.^29^ We cannot rule out such a type of shrinking mechanism, but we have verified that removing a C_2_ unit or a C atom for small cages is much more difficult, *i.e.* much less likely, than expanding and closing the cages.

## 2. Computational details

Amsterdam Density Functional code (ADF2011)^30,31^ was used for the electronic structure calculations and to optimize reactants, products, intermediates and transition states. The electronic density was provided by the local density approximation using Becke's gradient corrected exchange functional, and Vosko, Wilk, Nusair (VWN)^32^ parametrization for correlation, corrected with Perdew's functional (BP86).^33,34^ Electrons for carbon and titanium were described with Slater-type basis functions of triple-ζ + polarization quality. We have included scalar relativistic corrections by means of the zeroth-order regular approximation (ZORA) formalism. All calculations have also been performed including dispersion corrections.^35^ All the stationary points were fully characterized by computing the Hessian matrix.

Car–Parrinello molecular dynamics (MD) simulations were performed by means of the CPMD program.^36^ The description of the electronic structure was based on the expansion of the valence electronic wave functions in a plane wave basis set, which was limited by an energy cutoff of 40 Ry. The interaction between the valence electrons and the ionic cores was treated through the pseudopotential (PP) approximation (Martins–Troullier type). The functional by Perdew, Burke and Ernzerhoff (PBE)^37,38^ was selected as the density functional. We included nonlinear core corrections (NLCC) in the Martins–Troullier PP for the titanium atom. We used a fictitious electron mass of 800 a.u. The simulations were carried out using periodic boundary conditions in a cubic cell with a side length of 15 Å and a time step of 0.144 fs. The limited simulation time affordable by standard MD runs does not allow the observation of rare events such as thermally activated chemical reactions. For this reason, we employed the metadynamics technique,^39–41^ which is capable of efficiently reconstructing complex reaction mechanisms and providing the free energy profile, as demonstrated in previous applications.^42–47^ See the ESI† for more details.

## 3. Results and discussion

The experimental detection of the new family of endohedral metallofullerenes Ti@C_2*n*_ (2*n* = 26–46) is the starting point of this work. Common structural characterization techniques, which are key toward understanding EMF formation, are generally not applicable in gas-phase studies. Therefore, the first step of the investigation was to compute all the possible isomers for each of the Ti@C_2*n*_ detected. To do so, we designed the computational strategy taking into account all the factors involved in the stabilization of the EMFs: (i) the electronic structure of the empty cages; (ii) the formal charge transferred from the trapped metal atom to the carbon cage; and (iii) the interaction and position of the metal inside the carbon framework.

**Fig. 1:**
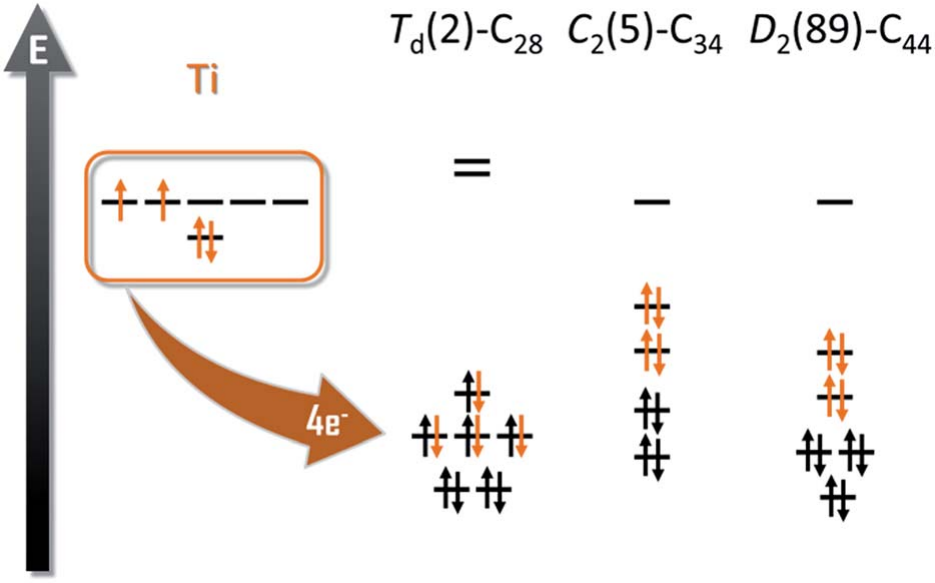
Molecular orbital diagrams for *T*_d_(2)–C_28_, *C*_2_(5)–C_34_ and *D*_2_(89)–C_44_. The four electrons formally transfer from the Ti atom to the carbon cage.

The number of isomers that can be built up rapidly increases from 1 single isomer for C_26_ to 271 for C_50_. For this reason, we decided to divide the whole family into two sets of structures, from C_26_ to C_34_ and from C_36_ to C_50_. We used the first set to determine if the ionic model, widely accepted for middle- and large-sized fullerenes, is valid for these smaller systems. It has been shown in previous works that the Ti atom formally transfers 4 electrons from its highest-occupied orbitals to the two lowest-unoccupied molecular orbitals of the carbon cage (Fig. 1).

### 3.1 Smallest Ti@C_2*n*_ systems (2*n* = 28–34): validation of the ionic model for small EMFs

This first set of systems includes a total of 17 isomers: 2 for C_28_, 3 for C_30_, 6 for C_32_ and 6 for C_34_ (Table 1). All isomers were constructed and labelled following the spiral algorithm.^48^ The relative energies of the anions are essentially maintained for the endohedrals (Table 1), showing the validity of the ionic model for such small EMFs as well. There are two possible isomers, of *D*_2_(1) or *T*_d_(2) symmetry, for the smallest endohedral fullerene cage, Ti@C_28_. It is known, however, from previous works, that the most stable isomer for the neutral, tetraanionic, and endohedral species is the *T*_d_(2)–C_28_ cage. The energy difference between the two C_28_ isomers increases from 17.5 kcal mol^–1^ for the neutral cages to more than 50 kcal mol^–1^ for tetraanions and EMFs. The *T*_d_(2)–C_28_ cage has an optimal electronic structure to accommodate the 4 electrons formally transferred from the Ti atom to the carbon cage (Fig. 1).

**Table 1:** Relative energies of the C_2*n*_ isomers (2*n* = 28–34) for neutral, tetraanions and endohedral fullerenes^*a*^

Cage	Isomer	C_2*n*_	C_2*n*_^4–^	Ti@C_2*n*_	*N*_p_ ^*b*^
C_28_	*D*_2_(1)	17.5	61.6	54.9	20
	*T*_d_(2)	**0.0**	**0.0**	**0.0**	**18**
C_30_	*D*_5h_(1)	49.7	58.6	62.9	20
	*C*_2v_(2)	2.6	17.6	20.1	18
	*C*_2v_(3)	**0.0**	**0.0**	**0.0**	**17**
C_32_	*C*_2_(1)	51.6	16.3	15.5	17
	*D*_2_(2)	62.5	39.4	35.1	18
	*D*_3d_(3)	66.1	29.1	31.0	18
	*C*_2_(4)	24.5	1.8	4.0	16
	*D*_3h_(5)	77.8	58.6	69.0	18
	*D*_3_(6)	**0.0**	**0.0**	**0.0**	**15**
C_34_	*C*_2_(1)	73.9	51.5	51.7	17
	*C*_s_(2)	25.4	4.6	5.9	15
	*C*_s_(3)	30.8	25.6	28.6	15
	*C*_2_(4)	15.3	7.9	8.0	15
	*C*_2_(5)	**0.0**	**0.0**	**0.0**	**14**
	*C*_3v_(6)	31.4	14.7	18.2	15

^*a*^Energies in kcal mol^–1^, at the BP86/TZP level; the lowest-energy EMFs have been highlighted in bold.

^*b*^ *N*_p_: number of fused pentagons or [5,5] bonds.

For the next largest cage formed by C_28_ + C_2_, *C*_2v_(3)–C_30_ is by far the lowest-energy isomer, at around 17 and 20 kcal mol^–1^ for the anion and endohedral systems, respectively. Upon further increasing the cage size by sequential additions of C_2_, the resulting C_32_ and C_34_ families show two isomers within a rather small range of energies. The lowest-energy Ti@C_32_ isomer is *D*_3_(6)–C_32_ with *C*_2_(4)–C_32_ only 4 kcal mol^–1^ higher in energy. For C_34_, the most stable system is *C*_2_(5)–C_34_ with *C*_s_(2)–C_34_ at almost 6 kcal mol^–1^. Calculated molar fractions at different temperatures, within both (i) the rigid rotor and harmonic oscillator approximation (RRHO) and (ii) the free encapsulation model (FEM), applicable when the internal metal atoms are able to rotate freely inside the carbon cage,^49,50^ predict Ti@*D*_3_(6)–C_32_ and Ti@*C*_2_(5)–C_34_ to be the most abundant isomers for the whole range of temperatures. The most stable isomers at 0 K for Ti@C_28_ and Ti@C_30_ were also found to be the most abundant ones at any temperature.

**Fig. 2:**
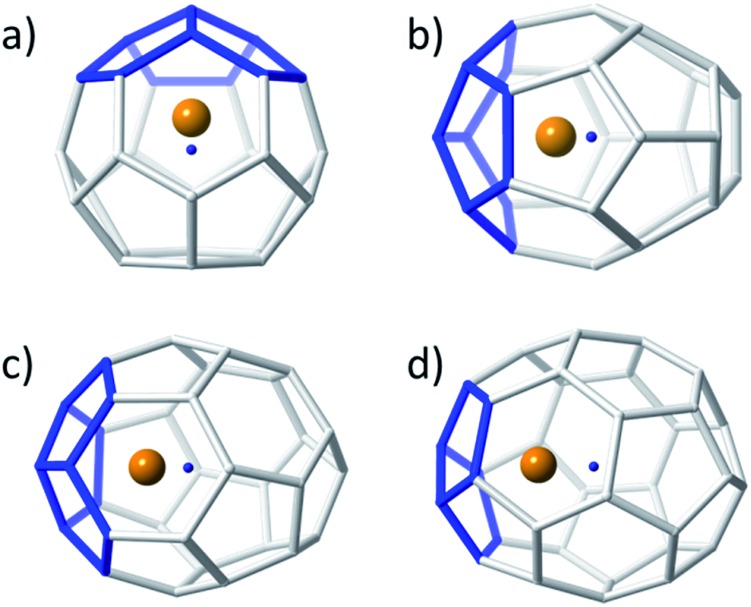
Ball and stick representations showing the displacement of the Ti atom from the centre of the cage for (a) Ti@*T*_d_(2)–C_28_, (b) Ti@*C*_2v_(3)–C_30_, (c) Ti@*D*_3_(6)–C_32_, and (d) Ti@*C*_2_(5)–C_34_. The Ti atom is represented as an orange sphere, the carbon cages as grey sticks and the centre of each cage as a small blue point. The [5,5,5] junctions nearest to the Ti atom are highlighted in blue.

Small non-IPR EMFs behave differently to middle-size and large systems. Larger EMFs that host a metal ion or cluster typically exhibit a different isomer than when the cage is empty. However, for the small Ti@C_2*n*_ systems, the hosting cage and the empty cage match. The so-called strain energy is the main factor that controls the relative stability in these systems. For this reason, those isomers with less structural tension, *i.e.* with fewer pentagon adjacencies, are the ones with the lowest energies. It is important to note that the formally transferred charge from the Ti atom to the carbon cage is preferentially located at the most strained regions, namely the [5,5] bonds and [5,5,5] junctions. Consequently, when encapsulated, the Ti ion prefers the most nucleophilic regions of the carbon cage, resulting in an off-centre shift to maximize the interaction with these regions (Fig. 2), as previously anticipated by other authors.^51^ The energy difference between the Ti-centered and the Ti-shifted systems is not negligible: about 37 kcal mol^–1^ for Ti@*T*_d_(2)–C_28_, 67 kcal mol^–1^ for Ti@*C*_2v_(3)–C_30_, 50 kcal mol^–1^ for Ti@*D*_3_(6)–C_32_ and 60 kcal mol^–1^ for Ti@*C*_2_(5)–C_34_.

### 3.2 Larger systems: Ti@C_2*n*_ (2*n* = 36–50)

The results obtained for the C_28_–C_34_ series clearly confirm the validity of the ionic model for small EMFs, as previously demonstrated for fullerenes with more than 60 atoms. When the number of carbon atoms increases, the number of isomers rapidly grows. For this second set of structures, the number of possible isomers is rather large (792), but still affordable to be computed at the DFT BP86/TZP level.

**Fig. 3:**
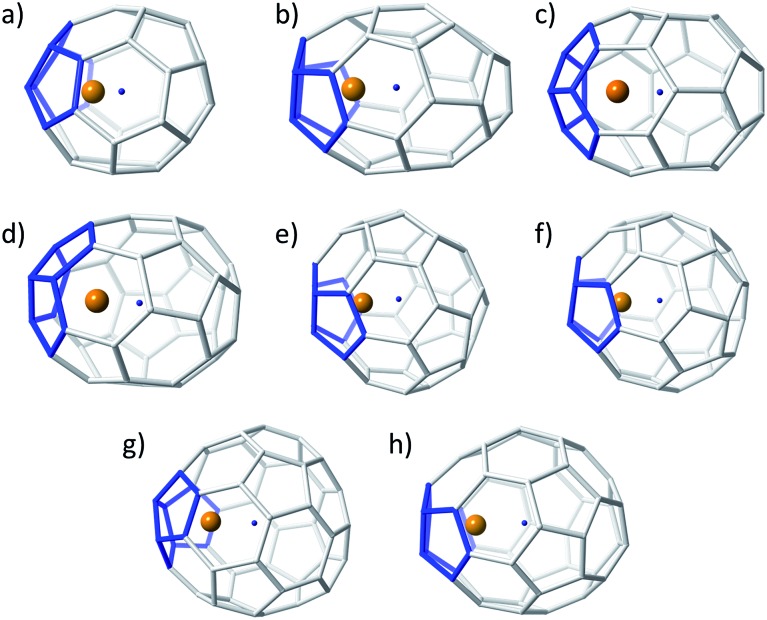
Ball and stick representations for the lowest-energy Ti@C_2*n*_ (2*n* = 36–50): (a) *D*_2d_(14)–C_36_, (b) *C*_2_(10)–C_38_, (c) *C*_s_(24)–C_40_, (d) *C*_1_(33)–C_42_, (e) *D*_2_(89)–C_44_, (f) *C*_2_(116)–C_46_, (g) *C*_s_(197)–C_48_, and (h) *C*_s_(266)–C_50_. Same colour codes as in Fig. 2.

For the lowest-energy tetraanions in a range of 30 kcal mol^–1^, an accurate exploration of the different possible positions of the Ti inside the cage was carried out to obtain the lowest-energy endohedral structures (Table 2). See the ESI† for the computational strategy that we propose for larger cages. Compared to the C_28_–C_34_ series, this second set of structures is found to exhibit a larger number of isomers in a smaller range of energies. Different trends were found to correlate with increasing EMF size. For example, two isomers, *D*_2d_(14) and *D*_6h_(15), were found to be degenerate in energy for C_36_ as neutral and tetraanionic species. However, isomer *D*_2d_(14) was somewhat more favoured when the Ti atom was encapsulated inside the cage (Fig. 3). For C_38_, although the *C*_2_(17) empty neutral cage was by far the lowest-energy isomer, *C*_2_(13) was the most stable cage computed as a tetraanion. For Ti@C_38_, cages *C*_2_(10) and *C*_2_(17) became competitive in terms of energy. Analogous results were observed for C_40_. The *D*_2_(38) cage was found to be the lowest-energy isomer, both in the neutral and tetraanion states. The most stable endohedral in this family was, however, Ti@*C*_s_(24)–C_40_. Several isomers in a small range of energies were found as well for C_42_. The *D*_3_(45) neutral empty cage was clearly the lowest-energy isomer. However, for C_42_^4–^ and Ti@C_42_ the cage *C*_1_(33) was the most stabilized. A different trend is observed in the case of C_44_, where only two isomers are found to be candidates within a large range of relative energies. The two cages, *D*_2_(75) and *D*_2_(89), showed small relative energy differences as neutrals, tetraanions and EMFs. Isomer *D*_2_(75) was the lowest-energy cage when the fullerene was computed as neutral, but isomer *D*_2_(89) became the most favorable when computed as a tetraanion and Ti@C_44_. For C_46_, we found again several isomers in a small range of relative energies. The neutral empty cages *C*_2_(109), *C*_1_(114) and *C*_2_(116) showed similar energies. Two of them, *C*_1_(114) and *C*_2_(116), were almost degenerate as tetraanions, the latter leading to the lowest-energy Ti@C_46_.

**Table 2:** Relative energies of the C_2*n*_ isomers (2*n* = 36–50) for neutral, tetraanions and endohedral fullerenes^*a*^

Cage	Isomer	C_2*n*_	C_2*n*_^4–^	Ti@C_2*n*_	*N*_p_ ^*b*^
C_36_	*C*_s_(8)	23.0	13.6	2.0	14
	*C*_2v_(9)	9.1	6.0	8.0	13
	*C*_2_(11)	12.8	10.2	5.3	13
	*C*_2v_(12)	7.8	7.2	4.4	13
	***D***_**2d**_ **(14)**	**0.6**	**0.0**	**0.0**	**12**
	*D*_6h_(15)^*c*^	0.0	0.0	4.3	12
C_38_	***C***_**2**_ **(10)**	**23.3**	**5.4**	**0.0**	**12**
	*C*_2_(13)	17.6	0.0	5.6	12
	*C*_1_(14)	24.3	7.4	6.7	12
	*C*_2_(17)	0.0	4.0	0.5	11
C_40_	***C***_**s**_ **(24)**	**23.9**	**3.4**	**0.0**	**11**
	*C*_s_(29)	16.7	5.4	6.0	11
	*C*_s_(31)	14.6	3.5	3.0	11
	*D*_2_(38)^*c*^	0.0	0.0	11.2	10
	*D*_5d_(39)	9.2	8.9	12.0	10
	*T*_d_(40)	37.8	1.1	7.5	12
C_42_	*C*_1_(32)	20.4	2.8	8.1	10
	***C***_**1**_ **(33)**	**19.6**	**0.0**	**0.0**	**10**
	*C*_s_(35)	23.3	6.9	4.5	10
	*C*_1_(39)	21.8	7.8	12.9	10
	*D*_3_(45)^*c*^	0.0	1.3	3.3	9
C_44_	*D*_2_(75)	0.0	7.2	5.0	8
	***D***_**2**_ **(89)**	**1.8**	**0.0**	**0.0**	**8**
C_46_	*C*_3_(94)	27.5	9.9	5.5	9
	*C*_2_(109)	0.0	9.3	14.9	8
	*C*_1_(114)^*c*^	1.9	0.0	7.9	8
	*C*_3_(115)	22.9	9.9	12.0	9
	***C***_**2**_ **(116)**	**2.5**	**0.9**	**0.0**	**8**
C_48_	*C*_2_(171)	0.0	7.7	10.7	7
	*C*_1_(196)^*c*^	2.0	1.3	5.4	7
	***C***_**s**_ **(197)**	**6.2**	**3.6**	**0.0**	**7**
	*C*_2_(199)^*c*^	0.8	0.0	4.2	7
C_50_	*C*_2_(260)	24.0	4.6	8.0	6
	*C*_2_(263)	10.5	7.2	11.7	6
	***C***_**s**_ **(266)**	**7.3**	**1.6**	**0.0**	**6**
	*D*_3_(270)	0.0	5.2	7.9	6
	*D*_5h_(271)	2.1	0.0	5.5	5

^*a*^Energies in kcal mol^–1^; the lowest-energy EMFs have been highlighted in bold.

^*b*^ *N*_p_: number of fused pentagons or [5,5] bonds.

^*c*^Isomers that become the most abundant at higher temperatures (see Fig. 5).

Similar results were obtained for C_48_ and C_50_. Isomers *C*_2_(171)–C_48_ and *C*_2_(199)–C_48_ were the lowest-energy empty cages, *C*_2_(199) being favoured in the tetraanionic state and *C*_s_(197) as Ti@C_48_. Finally, for C_50_, there was again a change in the relative stability depending on the charge state of the system. The lowest-energy isomers were *D*_3_(270) for neutral cages, *D*_5h_(271) for tetraanions and *C*_s_(266) for Ti@C_50_.

To explain these changes in the relative stabilities between the neutral cages, the tetraanions and the endohedrals, different factors have to be considered. The strain energy, which is a trend-setting factor for the smallest systems, is not as important when the size of the cage increases. Therefore, when the relative energies between isomers are small, the formal charge transfer and the interaction between the Ti atom and the carbon framework can reverse the relative stabilities of some isomers, as occurs for larger metallofullerenes.

**Fig. 4:**
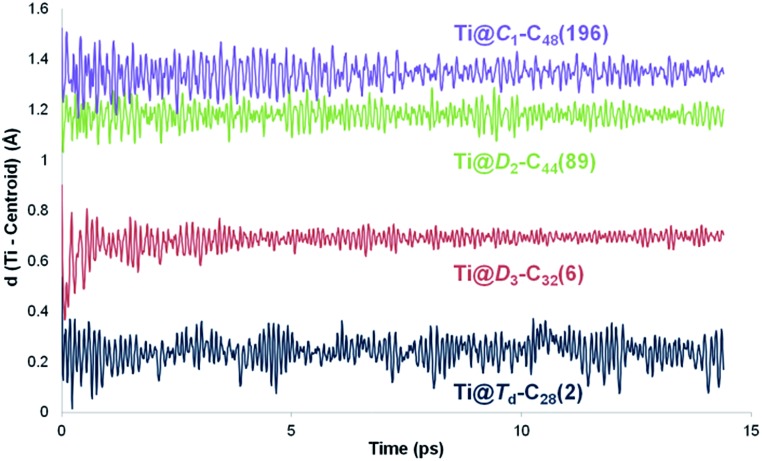
Variation of the distance between the center of the cage and the titanium atom (in Å) along 14 ps Car–Parrinello MD trajectories for Ti@*T*_d_–C_28_(2), Ti@*D*_3_–C_32_(6), Ti@*D*_2_–C_44_(89) and Ti@*C*_1_–C_48_(196).

The position of the metal inside of the cage is known to affect the properties of metallofullerenes.^6^ Off-centre displacement of the Ti atom inside these C_2*n*_ larger cages was confirmed by means of Car–Parrinello MD simulations, following the work of other authors.^52,53^ Oscillations up to 0.3 Å of the Ti atom around the optimal position, *i.e.* near the pentalene motifs, were observed for 14 ps trajectories at room temperature (Fig. 4). When the size of the cage increased, the distance between the titanium atom and the centre of the cage was also larger; the amplitudes of the oscillations did not change significantly though, indicating that Ti was rather fixed in a position near the adjacent pentagons. Oscillations with amplitudes, in some cases, larger than 0.7–0.8 Å were observed for 14 ps trajectories at 2000 K (Fig. S1†). For some cages, the Ti atom remained rather fixed, oscillating around its optimal position. In other cases, it was able to overcome the energy barrier to move to an equivalent position inside the cage within a few ps of simulation. From these results, we infer that Ti motion inside the C_2*n*_ cages is not negligible at the temperature of formation of fullerenes, but it does not move as completely freely as metal ions in larger IPR carbon cages.

**Fig. 5:**
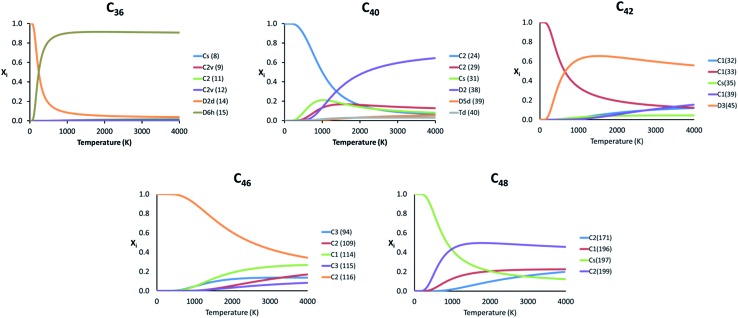
Representation of the molar fraction (*x*_i_) for the competitive isomers of the C_36_, C_40_, C_42_, C_46_ and C_48_ families using the RRHO approximation.

Because the relative energy between the endohedrals is rather small in many cases and they are synthesized at high temperatures, we have evaluated whether the effect of the temperature can reverse the stability of some isomers using the RRHO approximation and also the FEM model. As explained before, the Ti atom in Ti@C_2*n*_ systems moves appreciably, but not freely inside the fullerene. Therefore, the real behaviour of these systems should be at any point in between the two RRHO and FEM approximations. Fig. 5 shows the molar fractions (RRHO) of the most stable isomers for each Ti@C_2*n*_ family (2*n* = 36, 40, 42, 46 and 48) in the temperature range up to 4000 K. Inversions in the isomer populations were found at around 1000 K or even below for cages C_36_, C_40_, C_42_ and C_48_.

For the rest of the cages, there were no inversions in the molar fractions, that is, the lowest-energy isomers at 0 K were predicted to be the most abundant species in the whole range of temperatures. Similar results were obtained when computing the molar fractions using the FEM model (see Fig. S2†); the two most abundant isomers at *T* > 2000 K are predicted to be the same for almost all the families.

To sum up, whereas for smaller systems (2*n* < 36) the empty C_2*n*_ and endohedral Ti@C_2*n*_ match, when the number of carbon atoms increases, several isomers have similar stabilities and the most abundant EMFs are usually different from those predicted for the empty cages, as happens for fullerenes with more than 60 carbon atoms. The effect of the temperature was found to be relevant to predict the most abundant isomer for several Ti@C_2*n*_ families.

### 3.3 The Ti@C_26_ to Ti@C_28_ transformation: free energy profile and Car–Parrinello simulations

**Fig. 6:**
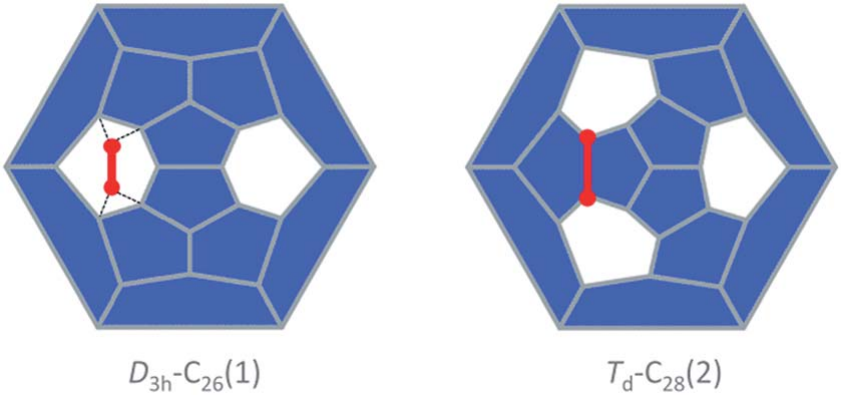
Schlegel representations of *D*_3h_–C_26_(1) and *T*_d_–C_28_(2) showing that the latter can be obtained from the former by ingestion of a C_2_ unit (red) as proposed originally by Endo and Kroto.^54^ Insertion does not take place in a single step, but through several intermediates (see text and Fig. 7).

The study of fullerene growth is focused on the direct C_2_ ingestion and, in few cases, subsequent structure rearrangements. The C_2_ molecule has been confirmed to play the most important role in fullerene growth, as deduced from experimental and theoretical results. Kroto and co-workers, within their Closed Network Growth (CNG) mechanism, proposed two possible paths to form C_62_ from C_60_ and C_2_ insertion.^27^ Both paths were investigated here for the transformation of Ti@C_26_, the smallest EMF detected so far, to Ti@C_28_, which could be the very first step in fullerene-to-fullerene conversion. In addition, this step can be accurately modelled and provides understanding for the transformations that take place for larger species. We found that direct C_2_ ingestion into the only possible isomer of Ti@C_26_ proceeds *via* a series of intermediates. The C_2_ molecule can be inserted into one of the three equivalent hexagons that *D*_3h_–C_26_(1) has (Fig. 6).

**Fig. 7:**
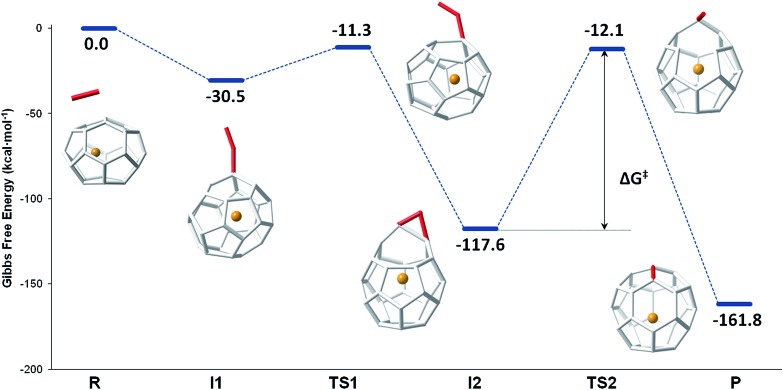
Gibbs free energy profile at 1000 K (in kcal mol^–1^) for the formation of Ti@C_28_ from Ti@C_26_ and C_2_ ingestion.

The mechanism proposed by Kroto and co-workers is based on a C_2_ molecule reacting with one [5,5,6] carbon atom of the selected hexagon, forming a first intermediate **I1**, in which the C_2_ unit is attached to the Ti@C_26_ surface through one C atom (see Fig. 7).

In this first step, no transition state (TS) is found. The Gibbs free energy difference between **I1** and the reactants is –31 kcal mol^–1^, so C_2_ attachment is a favourable process. From the corresponding C_62_ intermediate, Kroto and co-workers proposed two pathways to reach the same product. In the current work, we focused on one of them, which was shown to have the most stable intermediate. This second intermediate **I2** contains a seven-membered ring and it is characterized by its huge stability. A transition state **TS1**, in which the attached C_2_ unit is forming a C–C–C angle of 134.3 degrees, was found to connect **I1** and **I2**. The barrier to overcome this transition state is only 19 kcal mol^–1^. The final step corresponds to the closure of the cage, where C_2_ is finally inserted in the fullerene cage forming two fused pentagons in the original hexagon, as proposed by Endo and Kroto.^54^ The free energy barrier to overcome this transition state **TS2** is 106 kcal mol^–1^ and the Gibbs free energy difference for this last step is –44 kcal mol^–1^. Although this barrier is rather large, it is rather easily overcome at the temperatures at which fullerenes are formed (>1000 K). The entire insertion process is clearly exergonic (Fig. 7). No significant differences existed in the free energy profile if dispersion corrections were included or not.

The second path proposed by Kroto and co-workers was also studied.^27^ The only difference with respect to the mechanism explained before was that a second intermediate with a higher energy was found, **I2′**. In fact, for the Ti@C_28_ system under study, this stationary point was not a true intermediate, but a transition state that connected **I2** and its symmetrical structure (see Fig. S3†).

Gibbs free energy profiles at different temperatures between 1000 and 3000 K have been calculated to simulate the conditions under which fullerenes are formed. Similar shapes for the free energy profiles are obtained at different temperatures. With increasing temperature, however, the profiles are shifted to higher free energies with respect to the reactants, *i.e.* less exergonic, mainly due to the negative entropy change associated with the first step from reactants to **I1** (Fig. S4†).

**Fig. 8:**
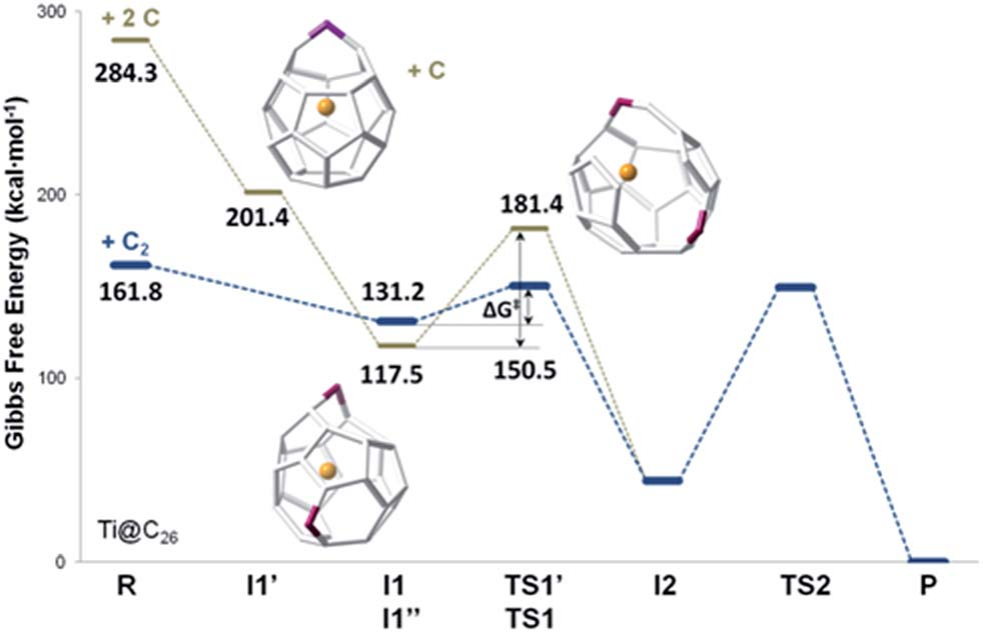
Free energy profiles at 1000 K (in kcal mol^–1^) for the formation of Ti@C_28_ from Ti@C_26_. The profiles for the C_2_ and the two successive C atom insertion mechanisms are represented with blue and grey lines, respectively.

We have also studied the successive insertion of atomic carbon proposed by Kroto and co-workers as a second possible cage growth process for empty fullerenes.^27^ Two carbon atoms were inserted separately into Ti@*D*_3h_–C_26_ to form the Ti@C_26_CC intermediate (Fig. 8). The first carbon ingestion to a [5,5,6] carbon atom led to intermediate Ti@C_26_C **I1′**, which presented a heptagon. There was no transition state in this step and the relative free energy at 1000 K with respect to the reactants was –83 kcal mol^–1^. The subsequent C insertion took place on another [5,5,6] carbon atom and led to intermediate Ti@C_26_CC **I1′′**, which was shown to have two heptagonal rings (Fig. 8). The free energy of formation of **I1′′** with respect to **I1′** is –84 kcal mol^–1^. Intermediate **I1′′** showed at 1000 K a somewhat lower free energy, 14 kcal mol^–1^, than the first intermediate from the C_2_ insertion mechanism **I1**. Once Ti@C_26_CC **I1′′** was formed, structural rearrangement to form **I2**, the second intermediate found for C_2_ insertion, might take place through transition state **TS1′** with a free energy barrier of 64 kcal mol^–1^ at 1000 K. The last step from **I2** to products was the same as for the C_2_ insertion mechanism. Both paths are compared in Fig. 8.

Besides the careful inspection of the potential energy surface and the free energy surfaces at different temperatures, we have gone beyond static analysis by simulating the ingestion of a C_2_ molecule into the Ti@C_26_ cage using Car–Parrinello molecular dynamics. The CNG mechanism, namely those structures shown in Fig. 7, was followed until **I2**, but any of the many simulated trajectories could overcome the second barrier associated with **TS2**. **I2** is a rather stable intermediate and could not evolve to the product at the short time scale of our simulations (tens of ps). In fact, this was basically because the barrier to reach the product is around 100 kcal mol^–1^ at 1000 and 2000 K. In some trajectories, we observed geometries near **TS2**, but the system had not enough energy to overcome the barrier and reach the product. As a consequence, we deemed it necessary to accelerate the dynamics at that point to observe such a rare event within our limited timescale of few ps. The metadynamics method was used, which also allowed us to estimate the free energy barrier for the rare event.^39–41^ Several successful metadynamics runs at 1000 K using different initial conditions and different sets of collective variables, coordination numbers of the atoms involved in the bond formation or some of their C–C distances (see ESI†), provided an estimation for the free energy barrier of 104 kcal mol^–1^, in good agreement with the results obtained from the static electronic structure calculations using the ideal gas and RRHO approximations to take into consideration temperature effects (Table 3).

**Table 3:** Reaction energies and free energies for the Ti@C_2*n*_ + C_2_ → Ti@C_2*n*+2_ processes^*a*^

Process	Δ*E*(P)^*b*^	Δ*E*(**I2**)^*c*^	Δ*E* ^‡,^ ^*d*^	Δ*G*(P)^*e*^	Δ*G*(**I2**)^*e*^	Δ*G* ^‡,^ ^*e*^	Δ*G*(P)–Δ*G*(**I2**)^*e*^
26 → 28	–207.4	–130.5	79.2	–161.8	–117.6	105.5	–44.2
28 → 30	–175.0	–115.8	83.6	–124.9	–72.3	83.7	–52.6
30 → 32	–190.7	–132.9	83.6	–130.4	–88.8	78.5	–41.6
32 → 34	–203.3	–122.5	61.6	–152.6	–81.3	63.5	–71.3
34 → 36	–197.6	–109.8	62.9	–153.1	–72.7	63.8	–80.4
36 → 38	–203.0	–115.5	34.5	–150.2	–73.1	41.9	–77.1
38 → 40	–198.8	–121.2	50.9	–153.4	–80.0	52.1	–73.4
40 → 42	–214.1	–126.3	50.0	–159.7	–78.7	50.2	–81.0
42 → 44	–218.8	–124.6	52.6	–172.3	–86.7	54.7	–85.6
44 → 46	–185.6	–100.7	62.4	–137.0	–58.5	60.0	–78.5
46 → 48	–201.4	–105.2	49.2	–151.2	–62.6	48.1	–88.6

^*a*^See Fig. 7 for the reaction profile. All energies are in kcal mol^–1^.

^*b*^Reaction energy for the process Ti@C_2*n*_ + C_2_ → Ti@C_2*n*+2_.

^*c*^Relative energy of the intermediate with respect to the reactants.

^*d*^Energy barrier corresponding to the step **I2** → product.

^*e*^Gibbs free energies at 1000 K.

The presence of an inert gas is required for the synthesis of fullerenes.^27^ Elucidating the role of this inert gas in EMF formation is important to gain insight about a likely formation mechanism. Perturbations to intermediate **I2** as a consequence of some external effects, such as, for example, collisions with the carrier gas (He or Ar atoms), as well as C_2_ molecules or C atoms, were also simulated (see the ESI† for more details on the computational settings). In a first and rougher approximation, the initial velocities of some C atoms, those involved in the cage closure, were increasingly modified as if they were activated by collisions with other atoms. At some point, we were able to observe the closure of the cage, that is, the formation of Ti@C_28_. We also simulated collisions between **I2** and one atom of the carrier gas (He or Ar), a C atom or a C_2_ molecule, at different initial conditions. Formation of the Ti@C_28_ cage was observed in most cases, but for collisions with a single C atom, the atom was incorporated into the carbon framework, either forming a C–C stick attached to the cage like an “octopus leg”, or forming a more closed Ti@C_29_ structure. Collisions with He atoms at velocities that were around 20 000 m s^–1^, *i.e.* kinetic energies around 9 eV, were seen to be successful at closing the Ti@C_28_ cage (*vide infra* and ESI†). Even though a very tiny fraction of He atoms will present such high kinetic energies at 2000–3000 K according to the Maxwell–Boltzmann velocity distribution, we have to keep in mind that conditions during fullerene formation are far from equilibrium.

### 3.4 Closed network growth (CNG) mechanism from C_26_ to C_48_

**Fig. 9:**
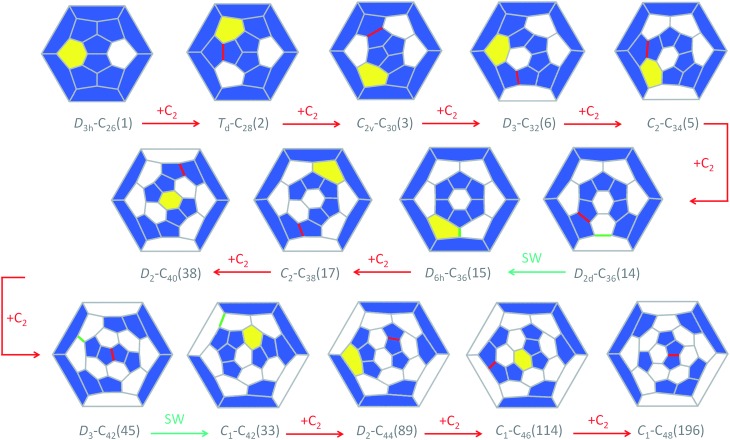
Schlegel diagrams connecting the most abundant isomers of each Ti@C_2*n*_ family. The hexagons where C_2_ inserts are highlighted in yellow, the formed C–C bonds are in red and the bonds that rearrange (Stone–Wales) are in green. Most of the lowest-energy isomers are linked through a C_2_ insertion and only a few of them are related by Stone–Wales transformations (cages C_36_ and C_42_). Minimization of pentagon adjacencies is not the sole criterion for EMF growth because for larger carbon cages the stabilization due to charge transfer can reverse the relative stabilities seen in neutral fullerenes.

The CNG mechanism is based on the insertion of a C_2_ unit to one hexagonal ring to form two fused pentagonal rings, in line with that proposed by Endo and Kroto in the early nineties.^54^ One consequence of this mechanism is that the new fullerene does not obey the isolated pentagon rule, but fullerenes smaller than C_60_, as analysed in this work, cannot obey this rule. In addition, the number of fused pentagons is progressively reduced as small fullerenes grow by C_2_ insertion (see the last column in Tables 1 and 2). According to the CNG formation of fullerenes, we propose a general mechanism for the growth of the cages from Ti@C_26_ to Ti@C_48_ based on successive C_2_ ingestions, in analogy to that recently proposed by Zhao and Nagase for empty C_2*n*_ fullerenes,^55^ based on the ideas of Fowler and Manolopoulos.^48^ The growth mechanism, represented in Fig. 9, relates the most abundant isomers for each Ti@C_2*n*_ family from 2*n* = 26 to 48 through simple C_2_ insertions and, in some cases, Stone–Wales (SW) rearrangements.

The first step, shown in Fig. 9, relates cages *D*_3h_–C_26_(1) and *T*_d_–C_28_(2). Once the C_2_ is ingested into the hexagon, two fused pentagons are formed (the new bond is shown in red in Fig. 9). Next, insertion of an additional C_2_ unit into the *T*_d_–C_28_(2) cage leads to the formation of *C*_2v_–C_30_(3). Further growth proceeds by inserting C_2_ molecules successively to give *D*_3_–C_32_(6), *C*_2_–C_34_(5) and *D*_2d_–C_36_(14), as for the hollow cages.^55^ This latter isomer is the second most abundant Ti@C_36_ after *D*_6h_–C_36_(15). These two cages are intimately related by a single SW rearrangement (the bond that rotates is marked in green in Fig. 9). Although SW transformations show rather large free energy barriers, 111 kcal mol^–1^ (4.8 eV) in the present case, they might be easily overcome at *T* > 1000 K. Moreover, previous theoretical and experimental reports find that for hollow C_60_ such rearrangements could be carbon-catalysed leading to atom exchange.^27,56,57^ For Ti@C_36_, the free energy barrier for the carbon-catalyzed transformation between cages *D*_2d_–C_36_(14) and *D*_6h_–C_36_(15) drops significantly to only 36 kcal mol^–1^ (1.6 eV), see the ESI† for more details. Thus, carbon-catalysed bond rearrangement could be a process involved in metallofullerene formation. It is important to remark here that the two cages *D*_2d_–C_36_(14) and *D*_6h_–C_36_(15) yield, after C_2_ insertion, the most abundant Ti@*C*_2_–C_38_(17) isomer. Ti@*D*_2_–C_40_(38) is then obtained and will evolve to Ti@*D*_3_–C_42_(45), the most abundant Ti@C_42_ isomer, after successive C_2_ ingestions. That species then grows to Ti@*D*_2_–C_44_(75), which is the second most stable Ti@C_44_ isomer, but with almost zero molar fraction for the full range of temperatures compared to the most abundant Ti@*D*_2_–C_44_(89). An alternative formation path to reach Ti@*D*_2_–C_44_(89) is through the Ti@*C*_1_–C_42_(33) precursor, formed by SW rearrangement of the initially formed Ti@*D*_3_–C_42_(45) species. C_2_ insertions into Ti@*D*_2_–C_44_(89) and Ti@*D*_2_–C_44_(75) lead to Ti@*C*_1_–C_46_(114) and Ti@*C*_2_–C_46_(109), respectively, which are among the most abundant Ti@C_46_ isomers at high temperatures, and are also related by a SW transformation. Additionally, Ti@*C*_1_–C_46_(114) is related to the most abundant Ti@*C*_2_–C_46_(116) isomer by a single SW rearrangement. Finally, the two most abundant Ti@C_48_ isomers, Ti@*C*_1_–C_48_(196) and Ti@*C*_2_–C_48_(199), are formed by direct C_2_ insertion into these Ti@C_46_ isomers. Therefore, the present mechanistic proposal relates the most abundant isomers of the Ti@C_2*n*_ series (2*n* = 26–48) by successive C_2_ insertions and a few number of SW rearrangements, in line with the CNG mechanism.

**Fig. 10:**
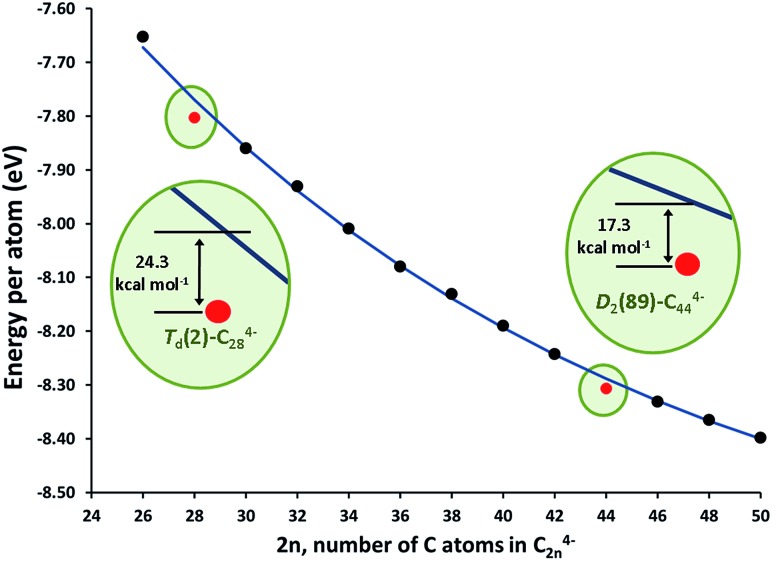
Energy per atom (in eV) for the lowest-energy C_2*n*_^4–^ isomers (2*n* = 26–50), black dots; and their fit to an exponential function, blue line. The energies for the *T*_d_–C_28_(2) and *D*_2_–C_44_(89) tetraanions are shown as red dots. The insets show the extra stability of these two isomers.

The stationary points and energetic profiles for these Ti@C_2*n*_ + C_2_ → Ti@C_2*n*+2_ processes were also characterized (2*n* = 28–46). The free energy profiles are totally equivalent to that found for 2*n* = 26 (see Table 3 and the ESI†). Analogous geometries for the intermediates, **I1** and **I2**, and transition states, **TS1** and **TS2**, were observed. In general, **I2** is much more stable than **I1**, and the barrier (**TS2**) that connects **I2** and the product was much higher than the one that connects **I1** and **I2** (**TS1**). These barriers (50–100 kcal mol^–1^), while somewhat high, are attainable at temperatures larger than 1000 K. Interestingly, lower free energy barriers are found for larger cages. Free energy barriers from Car–Parrinello metadynamics calculations are in good agreement with static calculations (see the ESI†). Reaction free energies at 1000 K are around –150 kcal mol^–1^ and show significant differences depending on the size of the cage. Low reaction free energies, in absolute values, might be related to those processes that have the most stable reactants. Lower exergonic reaction energies were found for the formation of Ti@C_30_ and Ti@C_46_. Therefore, Ti@C_28_ and Ti@C_44_ are predicted to be rather stable species. The energy per atom for the lowest-energy C_2*n*_^4–^ (and Ti@C_2*n*_) isomers with respect to the number of atoms is plotted in Fig. 10 (and S8†). As done by Popov for C_2*n*_^6–^ systems (2*n* = 68–98), the decrease of the energy as the cage becomes larger was fitted to an exponential decay.^58^ Cages *T*_d_–C_28_(2) and *D*_2_–C_44_(89) showed deviations of –24.3 and –17.3 kcal mol^–1^, respectively, with respect to the general behaviour, *i.e.* the fitted line in Fig. 10, confirming the enhanced stability of these two isomers. These results agree with the experimental FT-ICR-MS spectra obtained by Kroto and co-workers, where the largest peak intensities corresponded to Ti@C_28_ and Ti@C_44_.^28^

The transition states and the associated free energy barriers for non-catalyzed and carbon-catalyzed SW transformations on different Ti@C_2*n*_ (2*n* = 36 and 42) were also characterized. We find that the barriers for these particular catalyzed processes can be readily overcome at the high temperature required for metallofullerene synthesis.

### 3.5 CNG *vs.* shrinking mechanisms: insights from the free energy profiles and Car–Parrinello simulations

The insertion of C_2_ to small C_2*n*_ cages (2*n* = 26–48) is a thermodynamically favourable process, as the reaction free energies show (Table 3). The barriers for the CNG mechanism are easily attainable at the rather high temperatures of formation of fullerenes. As a consequence, the shrinking of these small C_2*n*_ cages by the loss of C_2_ units is a rather unfavourable endergonic process. Moreover, if cage shrinking were to take place through the same mechanism as ingestion (Fig. 7), significantly larger barriers of around 140 kcal mol^–1^ would have to be surmounted to reach **I2**. Therefore, from the free energy profiles at the formation temperatures of fullerenes, bottom-up growth by C_2_ ingestion is thermodynamically (reaction free energies) and kinetically (free energy barriers) more favourable than top-down shrinkage by C_2_ loss for these small Ti@C_2*n*_ fullerenes (2*n* = 28–48). Irle and Morokuma recently noted that large C_2*n*_ fullerenes (2*n* > 100 atoms) have a tendency to shrink whereas smaller fullerenes (2*n* < 100 atoms) tend to grow, as observed in molecular dynamics simulations at constant temperature and constant carbon density.^24^ Further, the carbon density was found to be a critical parameter to observe the growth or shrinkage of the cages.^24^

**Fig. 11:**
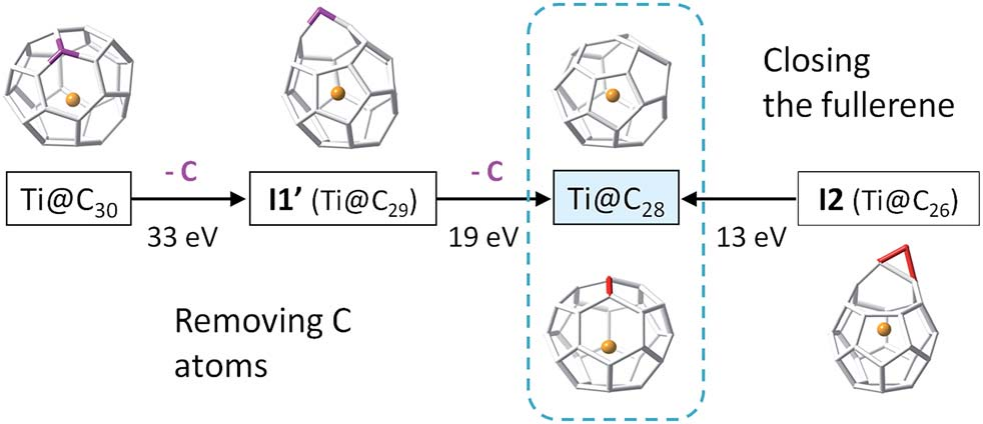
Collision kinetic energies involved in (i) the closure of **I2** Ti@C_28_ (right); and (ii) the shrinkage of Ti@C_30_ by successive removal of two C atoms (left). Closure of the endohedral metallofullerene requires kinetic energies above 13 eV, whereas the extrusion of single C atoms from the cage was always found to take place at much higher kinetic energies. These results correspond to simulations using initial structures optimized at 0 K. When initial distorted structures from MD at 2000 K were used, the kinetic energies needed to observe successful events were smaller (see the text).

To gain more insight about the bottom-up and shrinking mechanisms, we have simulated the collisions of several Ti@C_2*n*_ (2*n* = 28, 30, 44 and 48) with a buffer gas (He or Ar) at different initial conditions. In this way, we have accelerated the closure of the **I2** intermediate in bottom-up growth, and the extrusion of a single C atom for top-down shrinkage. Both are key steps in the two mechanisms, but they are very rare events that cannot be observed by standard Car–Parrinello molecular dynamics simulations at the time scale (tens of ps) that we can simulate. To observe successful cage shrinkage events, the required kinetic energy for the carrier gas is much higher than for bottom-up growth, in correlation with the values of the free energy barriers. For example, He atoms with kinetic energies of around 13 eV were needed to collide with the optimized **I2** structure to obtain Ti@C_28_, whereas He atoms with energies up to around 33 eV and greater were required to shrink Ti@C_30_ (Fig. 11). Interestingly, when distorted structures from MD simulations were considered as initial structures, the energy to close **I2** drops to 9 eV, while that to shrink Ti@C_30_ was mainly kept at 32 eV (see the ESI†). It should be pointed out here that more than one hundred and twenty MD runs, where He atoms collided with different velocities and orientations with respect to the carbon cage, were performed to simulate the shrinking mechanism for Ti@C_30_, and only seven of them led to the extrusion of a C atom from the closed fullerene (6%). On the other hand, about one hundred runs were done to simulate the bottom-up mechanism for the Ti@C_28_ system. In this case, around half of the collisions led to the closure of the fullerene from intermediate **I2**. Kinetic energies of ∼13 eV could seem at first sight rather huge – the free energy barrier to close the carbon cage is 4.5 eV. However, the He atom kept an energy of ∼3 eV after collision, so a total of ∼10 eV is transferred from the He to the cage. Energies much larger than the barrier were always found to be necessary for the event to take place. The extra energy (∼5 eV) is mainly dissipated through the vibrational modes. When distorted structures were considered, the collisions were seen to be more effective with appreciably less energy dissipation through vibrations (∼2 eV, see the ESI†). As the size of the cage increases, the barrier to close the cage decreases. Consequently, present results clearly support that larger cages can be formed at lower temperatures. For example, for C_48_ kinetic energies for the He atom of about 9 eV (8 eV for distorted structures) were needed to overcome a barrier of 2.1 eV (for more details see the ESI†). When collisions with much more energetic He atoms were simulated (of the order of keV, as in CID experiments), release of C_2_ units from Ti@C_2*n*_ systems (2*n* = 30 and 38) were observed, as found in experiments.^28^ Analogous results were obtained when the carrier gas was Ar, although in all simulations, larger kinetic energies for the colliding atom were needed (see the ESI†). Finally, we want to remark that in all the dynamics that simulate the shrinking process, we have found that the excess energy, dissipated essentially as vibrational energy, is much higher (>20 eV) than for the closing step of the carbon cage.

In summary, the free energy profiles for cage growth and shrinkage and the Car–Parrinello simulations of collisions with a carrier gas indicate that Ti@C_2*n*_ (2*n* = 28–48) should be mainly formed by bottom-up growth of smaller cages rather than top-down shrinkage of larger systems. These findings are in agreement with experimental results that show carbon cages grow in high-temperature environments of carbon vapor and helium gas, *i.e.* in fullerene synthesis conditions.

## 4. Conclusions

Recently, several mechanisms have been suggested to explain the formation of fullerenes. One of them involves the ingestion of a C_2_ unit by an already formed fullerene. In this article, we have explored this mechanism by means of static DFT and Car–Parrinello molecular dynamics calculations for the series of Ti@C_2*n*_ endohedral fullerenes, with 2*n* = 26 to 50. By comprehensive exploration of the most favourable isomers, the potential energy surfaces associated with successive C_2_ insertions and the topologies of the involved structures, we conclude the following:

(1) The insertion of a C_2_ unit to an already formed EMF is always an exothermic/exergonic process.

(2) The free energy barriers for each step are attainable considering the high temperature at which the processes occur.

(3) In striking contrast to larger EMFs (2*n* > 60), in which the empty cage is different from the optimal metallofullerene cage, non-IPR small endohedral fullerenes encapsulating a tetravalent ion often exhibit the same cage as empty fullerenes. While small metallofullerenes are well described by the ionic model, in these systems the strain energy should dominate over electrostatic repulsion, in contrast to larger, less strained EMFs with more than 70 cage atoms.

(4) The most abundant Ti@C_2*n*_ isomers are formally linked by direct C_2_ insertions, and in a few cases by additional Stone–Wales transformations.

(5) The presence of magic numbered EMFs, Ti@C_28_ and Ti@C_44_, in the mass spectra can be explained by the special electronic properties of these cages and their higher relative stabilities with respect to C_2_ reactivity.

(6) Car–Parrinello MD simulations show that after the attack of a C_2_ unit on one of the C atoms of the Ti@C_2n_ system, the formation of the second C–C bond that closes the cage is a very unlikely event at the time scale of our simulations, and must be accelerated using metadynamics or *via* an external collision, for example with He atoms that are present in the sample. Clearly, this is an extremely infrequent event that justifies the low abundance of small EMFs, only detected using special spectroscopic techniques.

To sum up, the present theoretical studies provide strong support for the CNG mechanism proposed to explain the empirically observed growth of small EMFs.^27^ As the size of the carbon cages increases, the free energy barriers associated with C_2_ insertion decrease and the collision kinetic energy required to close the cages also becomes smaller. The results imply that middle-sized endohedral metallofullerenes, 2*n* > 60, should have similar growth mechanisms, as preliminary studies seem to confirm. These results do not exclude shrinkage of fullerenes as an important process when the fullerene abundance is high and the carbon vapour density is low, as Irle and Morokuma have demonstrated.^24^ It might be noted that a recent study of the abundance estimates of large fullerenes in laser vaporization and carbon arc production, as well as some other studies, show that such estimates may have been overestimated resulting in erroneous conclusions. Experimental and theoretical studies on the growth of larger endohedral metallofullerenes are under way in our laboratories.
