# Supplementary material for: Small endohedral metallofullerenes: exploration of the structure and growth mechanism in the Ti@C2n (2n = 26–50) family
Source: Chem Sci. 2014 Sep 12;6(1):675–86. doi: 10.1039/c4sc02268h (PMC5590485; doi:10.1039/c4sc02268h)
Supplement: Supplementary file 1 [file SC-006-C4SC02268H-s001.pdf]

Electronic Supplementary Material (ESI) for Chemical Science.
This journal is © The Royal Society of Chemistry 2014

Supporting Information

Small Endohedral Fullerenes: Exploration of the Structure and Growth Mechanism in the Ti@C_{2n} (2n=26-50) Family

Marc Mulet-Gas,[†] Laura Abella,[†] Paul W. Dunk,[‡] Antonio Rodríguez-Fortea*[,†] Harry W. Kroto,*[,‡] and Josep M. Poblet*[,†]

[†] Departament de Química Física i Inorgànica, Universitat Rovira i Virgili, c/Marcel·lí Domingo s/n, 43007 Tarragona, Spain

[‡] Department of Chemistry and Biochemistry, 95 Chieftan Way, Florida State University, Tallahassee, Florida 32306, United States

Table of Contents

Fig. S1 Motion of Ti atom in Ti@C_{2n} cages at 2000 K S3

Fig. S2 Molar fractions using the FEM model S3

Fig. S3 Connectivity between **I2** and its symmetrical structure S4

Fig. S4 Free energy profiles at different temperatures S4

Fig. S5 Collective variables used in metadynamics S5

Table S1 Characteristics of the successful metadynamics S5

Fig. S6 Stone-Wales rearrangements profiles S6

Fig. S7 Free energy profile for a larger cage (2n = 42) S7

Fig. S8 Energy per atom for Ti@C_{2n} isomers (2n = 26-50) S7

Computational Settings for Collision Simulations S8

Fig. S9 He velocity profile in Car-Parrinello MD simulations S8

Table S2 Successful simulations of cage closure from **I2** and He atom S9

Table S3 Successful simulations of cage closure from **I2** and Ar atom S10

Table S4 Successful simulations of Ti@C_{30} cage shrinking with He S11

Table S5 Successful simulations of Ti@C_{30} cage shrinking with Ar S12

Fig. S10 He kinetic energies (KE) for collision processes S13

Tables S6 and S7 Average KE for closure and shrinkage of C_{2n} cages S13

Movie 1 Collision of He atom to Ti@C_{30} cage (shrinking) S14

Movie 2 Collision of He atom to **I2** (closure) S14

Fig. S11 AM1 vs DFT plots for C_{2n}^{4-} (2n=36-50) S15

xyz coordinates S16

(i) For the Ti@C_{26} + C_2 to Ti@C_{28} profile (R, P, intermediates, TSs) S16

(ii) The Ti@C_{2n} cages that appear in Fig. 9 S18

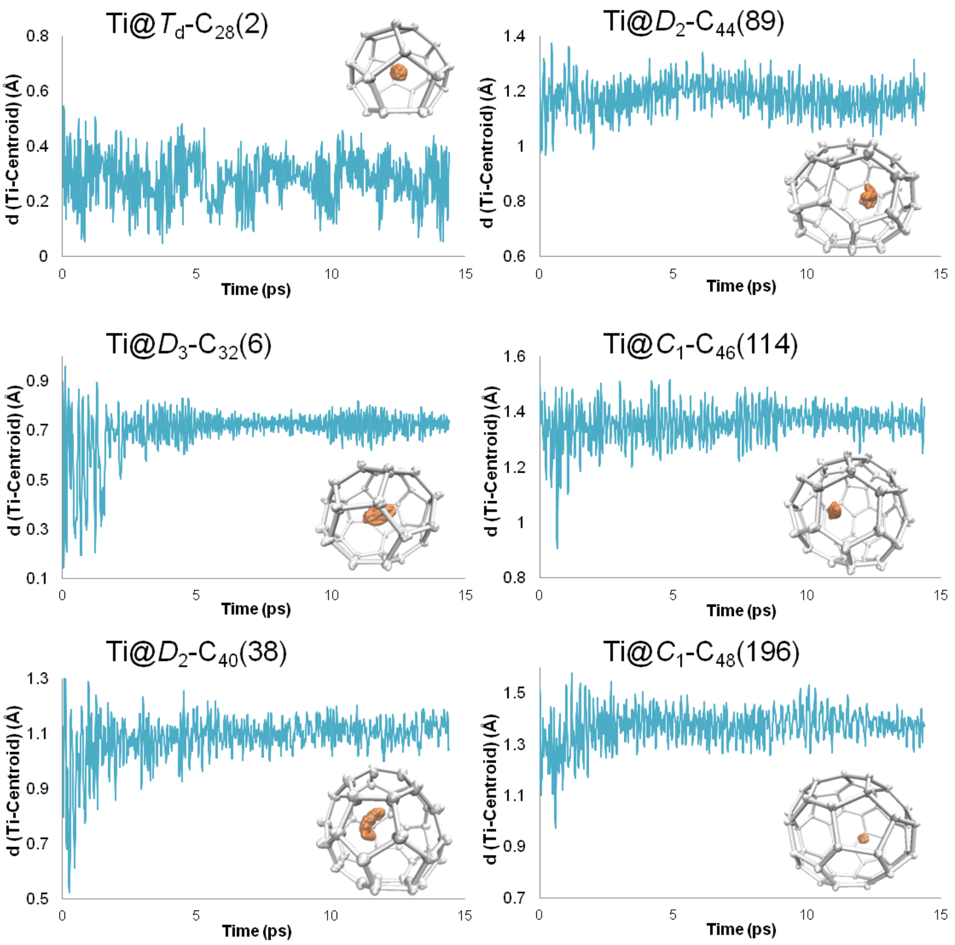

Fig. S1 Variation of the distance between the center of the cage and the Ti atom (in Å) along 14.4-ps Car-Parrinello MD trajectories for Ti@T_d-C_{28}(2), Ti@D_3-C_{32}(6), Ti@D_2-C_{40}(38), Ti@D_2-C_{44}(89), Ti@C_1-C_{46}(114), and Ti@C_1-C_{48}(196) at 2000 K.

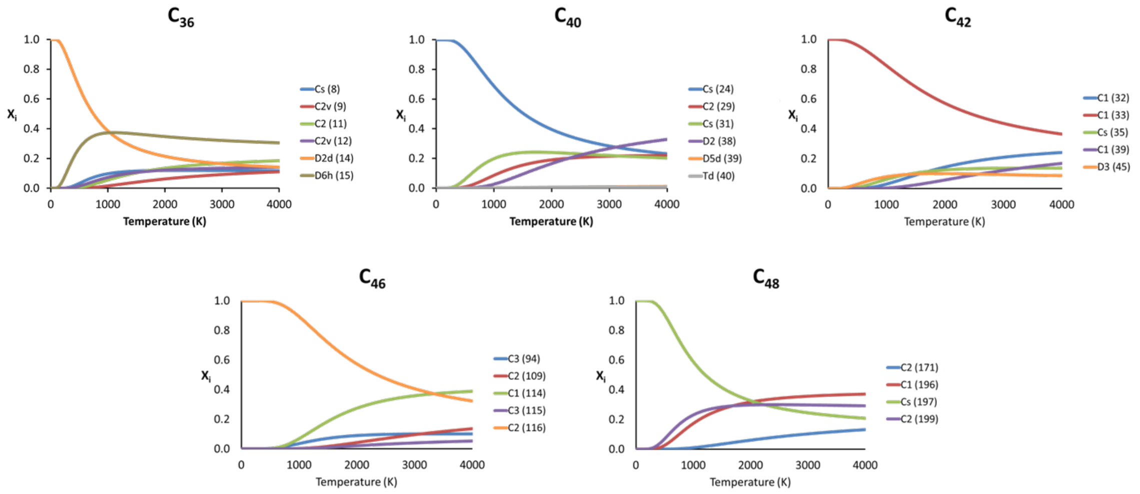

Fig. S2 Representation of the molar fraction (x_i) for the competitive isomers of C_{36}, C_{40}, $C_{4,}$ C_{46} and C_{48} families using the FEM model.

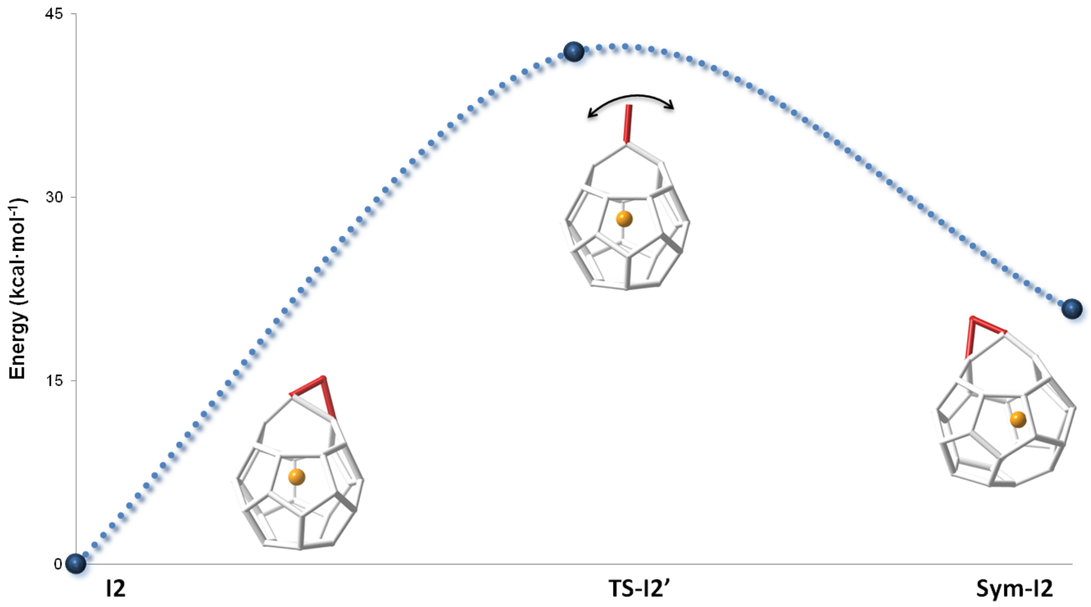

Fig. S3 Energy profile (in kcal·mol^{-1}) for the pathway that connects **I2** and its symmetrical structure, **Sym-I2**. The two structures are connected through transition state **TS-I2',** which shows a structure very similar to the second intermediate proposed by Kroto and co-workers in *Nature Commun.* **2012**, *3*, 855. The structures of **I2**, **Sym-I2**, and **TS-I2’** are also shown in the profile. For the **TS-I2** structure, the arrow describes the motion of the C atom in the normal mode with the imaginary frequency.

The energy of **Sym-I2** is higher than that of **I2** because the Ti atom is trapped in a local minimum inside the cage. Optimization of the position of Ti atom leads to the same structure and energy as for **I2**.

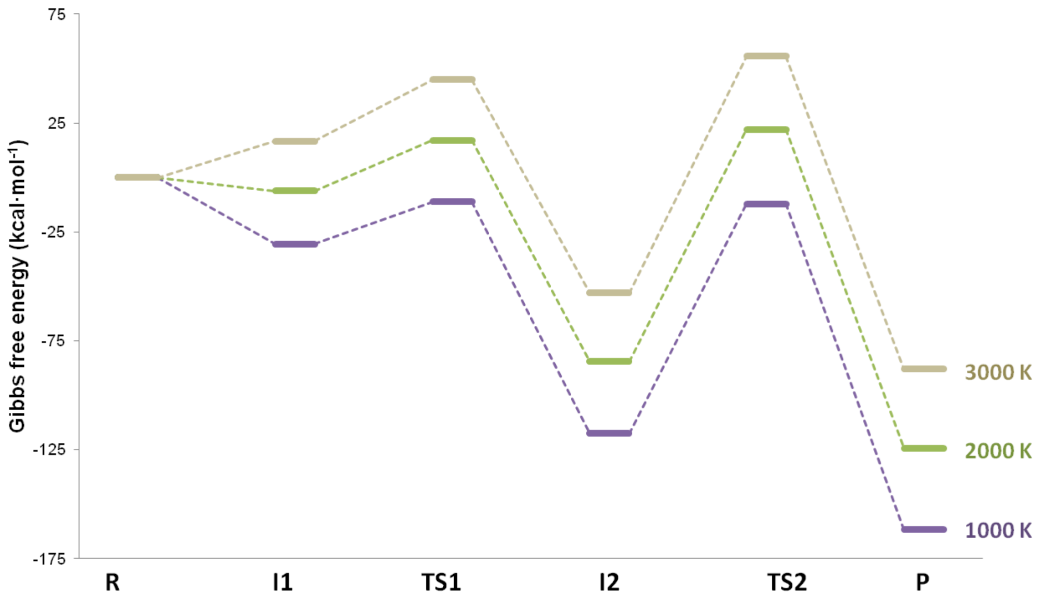

Fig. S4 Gibbs free energy profiles (in kcal·mol^{-1}) at different temperatures (1000, 2000 and 3000 K) for the formation of Ti@C_{28} from Ti@C_{26} and C_2.

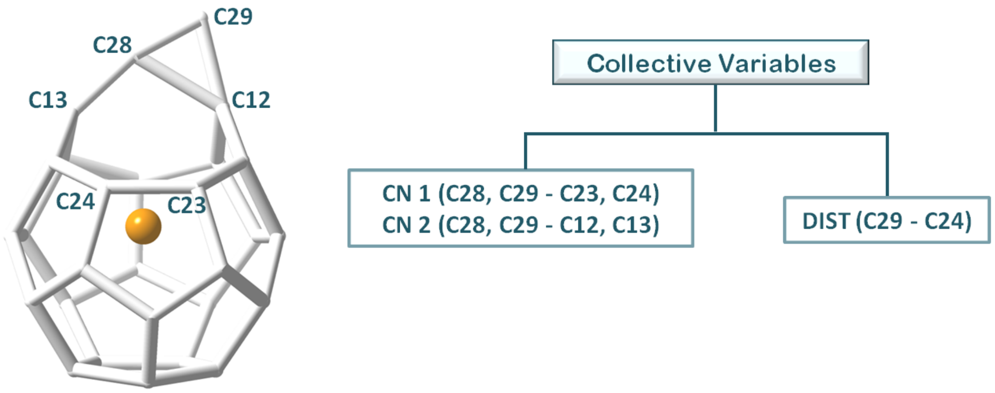

Fig. S5 Sets of collective variables (CV) used in different metadynamics at 1000 K. Carbon atoms involved in the collective variables are labeled in the intermediate **I2**. On one hand, two coordination numbers (CN) are used to perform the metadynamics. The first coordination number, CN 1, is involved in the [5,5] bond formation, and the second one, CN 2, follows the breaking of the triangular cycle. On the other hand, one C-C distance (DIST) is also used as CV. Carbon atoms involved in each set are indicated next to the type of the CVs.

Table S1 Successful metadynamics of the closure of Ti@C_{2n}. Values of the energy barrier (in kcal·mol^{-1}), type of collective variables (CV) and their k and M values, as well as height, Δ**s**, (in a.u.) and width, W, (in a.u.) of the gaussians.

2n → 2n + 2 (2n)	Barrier (kcal·mol^{-1})	CV	k[a]	M[b]	Δs	W (a.u.)
26	104	2 CN	5 / 2	100 / 30	0.05	0.0015
	125	2 CN	5 / 2	150 / 30	0.05	0.0015
	98	2 CN	7 / 2	150 / 30	0.05	0.0015
	90	2 CN	7 / 2	100 / 30	0.05	0.0015
	104*					
28	83	2 CN	7 / 2	100 / 30	0.05	0.0015
30	76	2 CN	10 / 2	150 / 30	0.05	0.0015
42	54	2 CN	3 / 2	150 / 30	0.05	0.0015
44	61	DIST	10	150	0.15	0.0020
46	50	DIST	7	150	0.15	0.0020

[a] The coupling constants (in Hartree((t)/(u.s.))^2). [b] The fictitious masses. Both determine the dynamics of the $\{s_\alpha\}$ in the CV-space.*Average of all the barriers shown in 26 → 28 process.

a

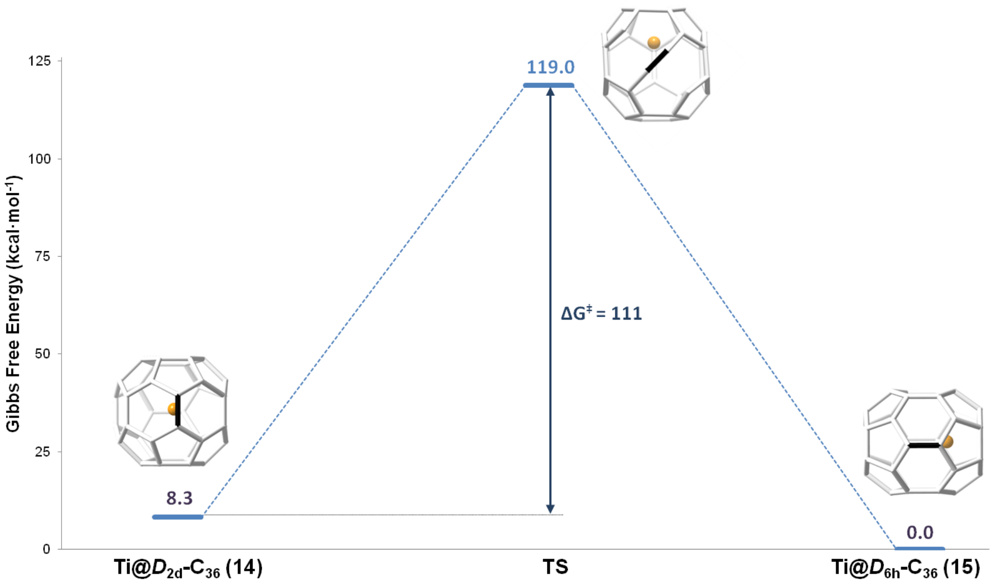

b

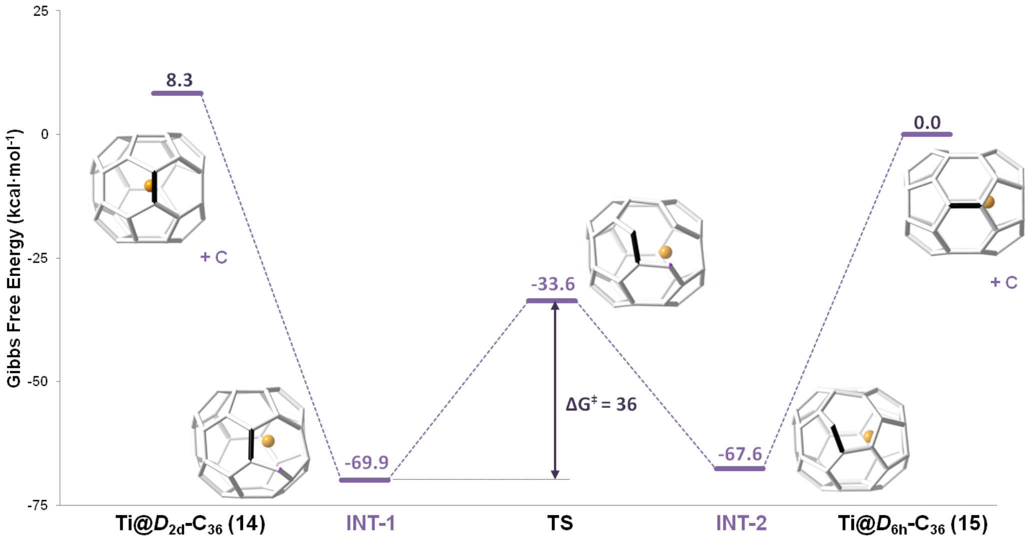

Fig. S6 Gibbs free energy profiles (in kcal·mol^{-1}) comparing a) the conventional Stone-Wales rearrangement, and b) the carbon-catalyzed bond rearrangement leading to atom exchange. Stone-Wales transformations for Ti@D_{2d}-C_{36}(14) isomer to Ti@D_{6h}-C_{36}(15) isomer. The conventional SW transformation free energy barrier is found to be 110.7 kcal·mol^{-1}, whereas the carbon-catalyzed bond rearrangement free energy barrier is found to be lower, 36.3 kcal·mol^{-1}. The insertion of a carbon atom in Ti@D_{2d}-C_{36}(14) cage leads to the first intermediate, **INT-1**, in the carbon-catalyzed bond rearrangement mechanism. Once the transition state is overcome, it reaches the second intermediate, **INT-2**. All the geometries for both mechanisms are shown in the figure.

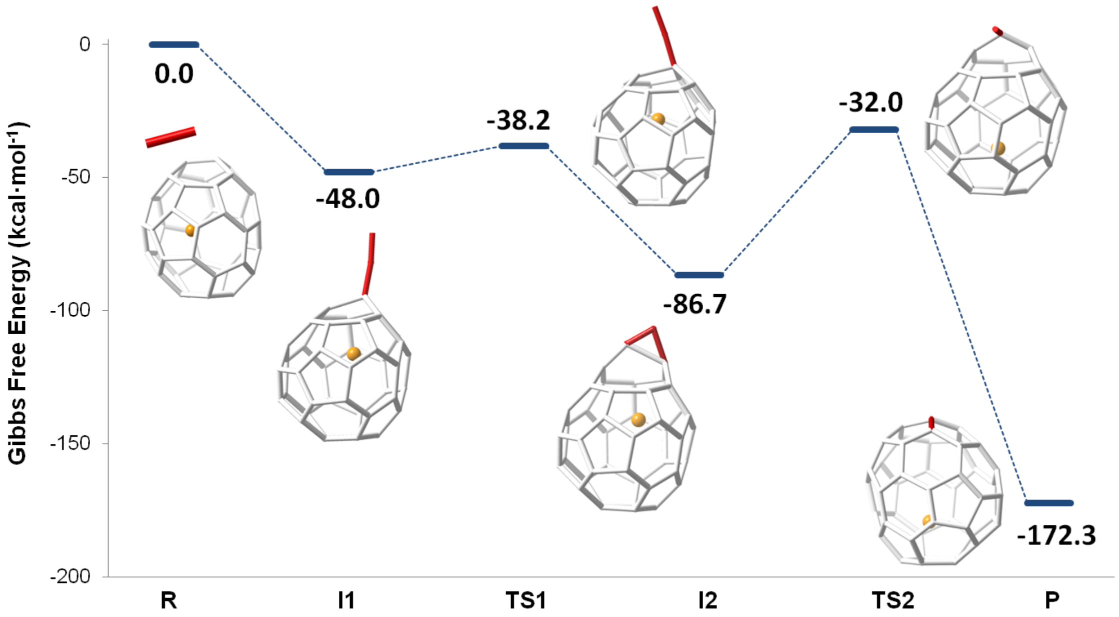

Fig. S7 Gibbs free energy profile at 1000 K (in kcal·mol^{-1}) for the formation of Ti@C_{44} from Ti@C_{42} and C_2 ingestion. Free energy barrier, **TS2**, is found to be 54.7 kcal·mol^{-1} in this mechanism. All the intermediates, **I1** and **I2**, and transition states, **TS1** and **TS2**, which are shown in the profile, are equivalent to those found for Ti@C_{26} to Ti@C_{28}.

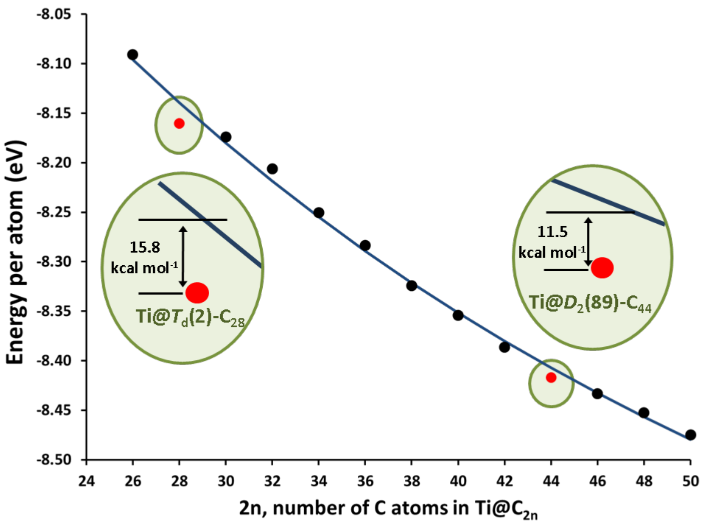

Figure S8. Energy per atom (eV) for the lowest-energy Ti@C_{2n} isomers (2n = 26-50), black dots, with respect to the number of C atoms. The energies for Ti@T_d(2)-C_{28} and Ti@D_2(89)-C_{44} cages are shown as red dots. The inset shows the extra stability of these two isomers.

Computational settings for simulations of collision processes. Collisions with C_2 molecules, or C, He or Ar atoms were simulated using Car-Parrinello Molecular Dynamics by modifying the initial velocities (both speed and direction of motion) of C_2, C, He or Ar and the total initial temperatures. These short simulations (around 1 ps) were done in the NVE ensemble.

In general, velocities of He atoms were found to be larger than Ar atoms for both processes growth and shrinkage (as expected for similar temperatures because He is much lighter than Ar). The same criterion for collisions with Ar and He was used. All kinetic energies or velocities shown in the following tables (Table S2, S3, S4 and S5) or in the text are taken arbitrarily from the black circle zone of each MD run (see Fig. S9); it is the velocity of the colliding atom, once stabilized, and just before the collision with the carbon cage.

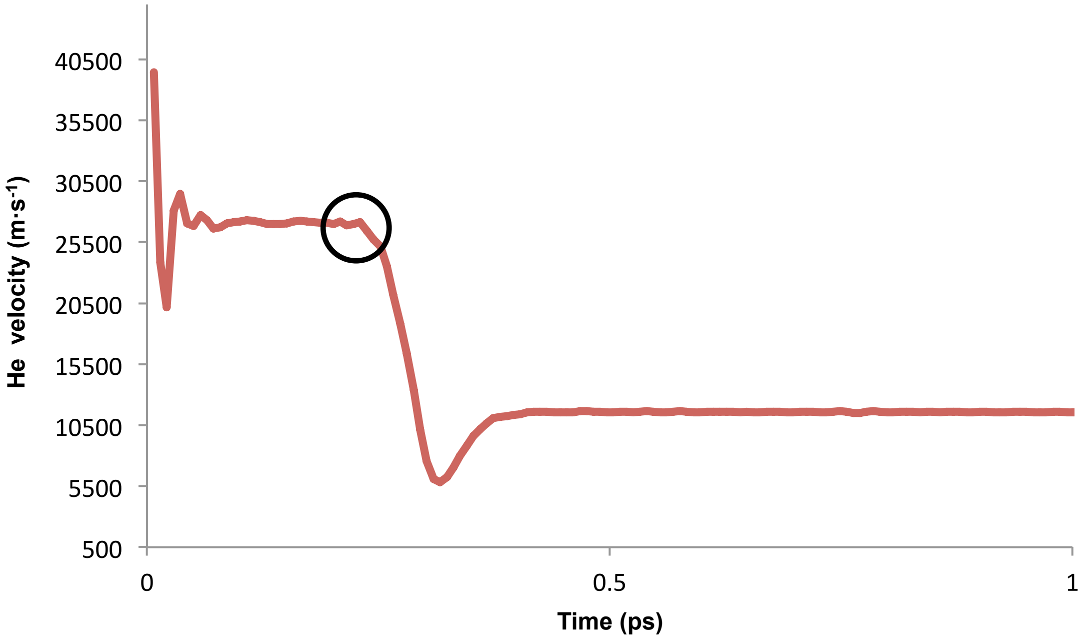

Fig. S9 Variation of He velocity (in $m \cdot s^{-1}$) during the Car-Parrinello MD simulation. The time/velocity of the collision is represented by the black circle.

Table S2 Successful Car-Parrinello MD simulations of the closure of Ti@C_{2n} as a consequence of collision between **I2** and He atom. Initial temperatures (Initial T) of the system for each MD run are shown in Kelvin. He velocity (in $m{\cdot}s^{-1}$) and kinetic energy (in eV) just before collision takes place, as well as the corresponding distance between He and C atoms (in Å) at that time, are shown. The initial structures used for the **I2** intermediates are those previously optimized at BP/TZP level. For 2n = 28 and 48, distorted structures of **I2** intermediates obtained form NVT dynamics at temperatures of 1000 K and 2000 K were also used as initial structures in the collision simulations.

I2 to Ti@C_{2n} (2n)	Initial T (K)	dist He-C (Å)	He velocity ($m{\cdot}s^{-1}$)	Kinetic Energy (eV)
**28**	11000	1.69	27600	15.8
(Optimized **I2**)	12000	1.64	28200	16.5
	13000	1.60	28600	17.0
	15000	1.75	33000	22.5
	12000	1.77	29000	17.4
	13000	1.71	29600	18.1
	15000	1.59	30600	19.4
	8000	1.74	25200	13.1
	8000	1.64	24700	12.6
	13000	1.60	30500	19.3
	8000	1.64	24700	12.6
	10000	1.71	27600	15.8
	9000	1.74	25200	13.1
	8000	1.70	26600	14.7
	10000	1.91	27700	16.0
	9000	1.70	26600	14.7
	10000	1.71	27800	16.0
	9000	1.96	27100	15.2
	10000	1.71	27700	16.0
	7500	1.83	24700	12.6
	9000	1.70	26600	14.7
**28**	5000	1.84	20695	8.9
(Distorted **I2**)	6000	1.82	22513	10.5
	8000	1.73	25167	13.1
	9000	1.61	25931	13.9
	6000	1.83	22524	10.5
	8000	1.73	25176	13.1
	8000	1.86	25350	13.3
	7000	1.74	23907	11.8
	8000	1.78	25237	13.2
	7000	1.86	24000	11.9
	7000	1.84	24000	11.9
	6000	1.82	22500	10.5
	7000	1.68	23800	11.7
	7000	1.68	23800	11.7
	8000	1.73	25100	13.1
	5000	1.85	20700	8.9
	6000	1.83	22500	10.5
**30**	10000	1.66	28000	16.3
	10000	1.74	28300	16.6
	9000	1.76	27200	15.3
	10000	1.67	28100	16.3
	10000	1.69	28200	16.5
	11000	1.75	29700	18.3
	12000	1.64	30500	19.3
	9000	1.78	27400	15.6
**44**	3000	2.23	20300	8.5
	5000	1.67	24700	12.6
	3000	1.89	19000	7.5
	5000	1.74	24500	12.4
	2000	2.01	16300	5.5
	3000	1.90	20100	8.4
	5000	1.75	24900	12.9
	2000	2.00	16400	5.6
	3000	1.89	20100	8.4
	2000	2.00	16700	5.8
	3000	2.00	20200	8.5
	2000	2.00	16600	5.7
	3000	1.98	20300	8.5
**48**	3000	2.04	21200	9.3
(Optimized **I2**)	5000	1.62	25900	14.0
	3000	1.81	20700	8.9
	5000	1.66	25600	13.6
	3000	1.87	20900	9.1
	3000	1.87	21000	9.1
	3000	1.80	20700	8.9
	4000	1.69	23400	11.4

**48**	3000	1.69	20000	8.3
(Distorted **I2**)	3000	1.69	20000	8.3
	3000	1.81	20700	8.9
	3000	1.81	20800	8.9

Table S3 Successful Car-Parrinello MD simulations of the closure of Ti@C_{2n} as a consequence of collision between **I2** and Ar atom. Initial temperatures (Initial T) of the system for each MD run are shown in Kelvin. Ar velocity (in $m \cdot s^{-1}$) and kinetic energy (in eV) just before collision takes place, as well as the corresponding distance between Ar and C atoms (in Å) at that time, are shown. Optimized **I2** structures are used as initial structures in the collision simulations.

I2 to Ti@C_{2n} (2n)	Initial T (K)	dist Ar-C (Å)	Ar velocity ($m \cdot s^{-1}$)	Kinetic Energy (eV)
**28**	7000	2.51	10200	21.5
	7000	2.51	10200	21.5

Table S4 Successful Car-Parrinello MD simulations of the shrinking of Ti@C_{30} as a consequence of collision with He atom. Initial temperatures (Initial T) of the system for each MD are shown in Kelvin. He velocity (in $m \cdot s^{-1}$) and kinetic energy (in eV) just before collision takes place, as well as the corresponding distance between He and C atoms (in Å) at that time, are shown. Optimized and distorted (NVT MD at 2000 K) geometries were used as initial structures in the collision simulations.

System	Initial T (K)	dist He-C (Å)	He velocity ($m \cdot s^{-1}$)	Kinetic Energy (eV)
**Ti@C_{30} to I1'**	26000	1.64	39700	32.7
(Optimized Ti@C_{30})	27000	1.61	39800	32.8
**Ti@C_{30} to I1'**	29000	1.58	39800	32.9
(Distorted Ti@C_{30})	30000	1.56	39700	32.7
	35000	1.60	38300	30.5
	26000	1.95	39000	31.6
	27000	1.91	39400	32.2
	28000	1.88	39700	32.7
	29000	1.84	40000	33.2
	30000	1.81	40300	33.7
	33000	1.72	40900	34.7
	32000	1.64	37800	29.7
	33000	1.62	37900	29.8
	29000	1.58	39800	32.8
	25000	1.99	38600	30.9
**I2 to I1'**	13000	1.90	31200	20.2
(Optimized **I2**)	15000	1.93	33000	22.6
	13000	1.71	31200	20.2
	15000	1.68	32400	21.8
**I2 to I1'**	11000	1.81	28700	17.1
(Distorted **I2**)	12000	1.73	29500	18.0
	13000	1.66	30100	18.8
**I1' to Ti@C_{28}**	12000	1.78	30200	19.0
(Optimized **I1'**)	13000	1.70	31000	20.0
	15000	1.56	32300	21.6
	15000	1.70	32400	21.8
**I1' to Ti@C_{28}**	14000	1.71	30700	19.6
(Distorted **I1'**)	11000	1.64	28100	16.3
	12000	1.77	28900	17.3
	10000	1.70	27600	15.8
	11000	1.61	28400	16.7
	12000	1.53	29100	17.5
	12000	1.56	28900	17.3
	11000	1.81	28500	16.8
	12000	1.53	29000	17.5
**I1 to Ti@C_{28}**	6000	1.81	22700	10.7
(Optimized **I1**)	7000	1.81	24900	12.9
	10000	1.83	28600	17.0
	6000	1.79	23000	11.0
	7000	1.73	24400	12.3
	10000	1.79	28400	16.7
**I1 to Ti@C_{28}**	7000	1.65	24000	12.0
(Distorted **I1**)	6000	1.76	22900	10.8
	4000	2.03	18800	7.3
	5000	1.97	20800	9.0
	6000	1.96	22700	10.7

Table S5 Successful Car-Parrinello MD simulations of the shrinking of Ti@C_{30} and Ti@C_{28} as a consequence of collision with Ar atom. Initial temperatures (Initial T) of the system for each MD are shown in Kelvin. Ar velocity (in $m{\cdot}s^{-1}$) and kinetic energy (in eV) just before collision takes place, as well as the corresponding distance between Ar and C atoms (in Å) at that time, are shown. Optimized structures are used as initial structures in the collision simulations.

System	Initial T (K)	dist Ar-C (Å)	Ar velocity ($m{\cdot}s^{-1}$)	Kinetic Energy (eV)
**Ti@C_{30} to I2**	17000	2.47	15600	50.4
	19000	2.43	16400	55.7
**Ti@C_{30} to I1**	13000	2.42	14100	41.2
**I2 to I1'**	12000	2.55	13700	38.9
	13000	2.51	14200	41.7
	15000	2.48	15300	48.5
	17000	2.50	16300	50.5
	11000	2.50	13100	35.5
	12000	2.40	13700	38.9
	13000	2.42	14200	41.7
	15000	2.42	15300	48.5
	17000	2.48	16300	55.0
**I1 to Ti@C_{28}**	3000	2.53	6700	9.29
	4000	2.46	7750	12.4
	5000	2.51	8760	15.9
	6000	2.53	9630	19.2
	7000	2.46	10400	22.4
	8000	2.43	11100	25.5
**I1' to Ti@C_{28}**	13000	1.78	13800	39.4
	15000	2.02	15000	46.6
**Ti@C_{28} to I2**	13000	2.46	13600	38.3
**I2 to I1'**	12000	2.56	13100	35.5
	13000	2.55	16300	38.3
	15000	2.55	14700	44.7
	12000	2.53	12900	34.5
	13000	2.49	13500	37.7
	15000	2.46	14500	43.5
**I1' to Ti@C_{26}**	12000	2.62	12800	33.9

a)

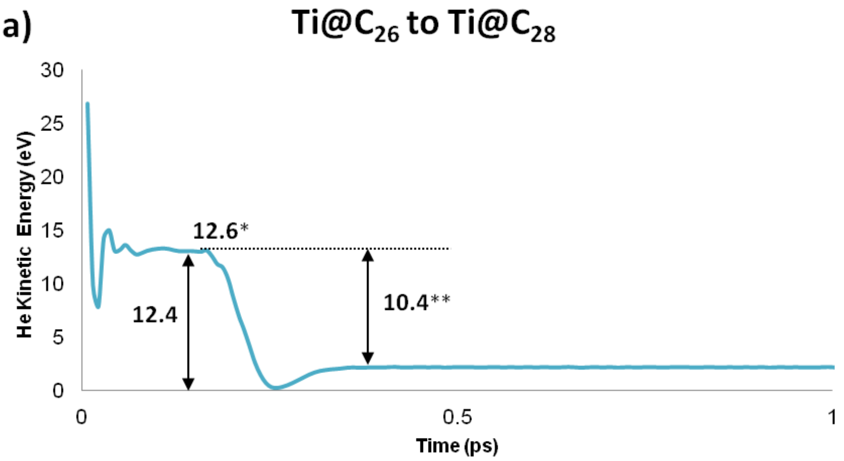

b)

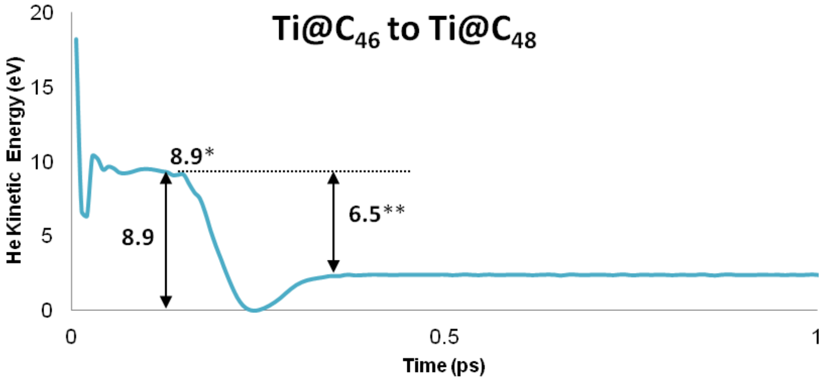

Fig. S10 Kinetic energy (in eV) of the He atom during the MD run simulating the collision to **I2** in a) Ti@C_{26} to Ti@C_{28} system, and b) Ti@C_{46} to Ti@C_{48} system. The average of these kinetic energies (KE) are listed in Table S6. * Kinetic energy for the He atom required to close the fullerene cage. ** Kinetic energy transferred to the carbon cage as a consequence of the collision.

Table S6 Average He kinetic energies (in eV) for closure of different Ti@C_{2n} from the collision of **I2** and He atom. Minimum He kinetic energies (in eV) required to have a successful event are found in parenthesis.

I2 to Ti@C_{2n} (2n)	KE He* (eV)	KE transferred** (eV)	$\Delta G^{\ddagger\,a)}$ (eV)	Extra KE b) (eV)
28	14.2 (12.6)	11.9 (10.4)	4.5	7.4 (5.9)
28c)	11.6 (8.9)	9.4 (6.9)	4.5	4.9 (2.4)
30	16.4 (15.3)	13.2 (12.1)	3.6	9.6 (8.5)
44	6.5 (5.5)	4.7 (4.4)	2.4	2.3 (2.0)
48	9.1 (8.9)	6.5 (6.2)	2.1	4.4 (4.1)
48c)	8.5 (8.0)	6.3 (6.2)	2.1	4.2 (4.1)

* Kinetic energies (in eV) for the He atom required to close the cage. ** Kinetic energy (in eV) transferred from the He atom to the carbon cluster. a) Gibbs free energy barrier (in eV) for each system. b) Extra kinetic energy, KE transferred - $\Delta G^{\ddagger}$, (in eV) mainly dissipated as vibrational energy. c) Distorted structure from NVT MD at 2000 K as initial structure.

Table S7 Average He kinetic energies (KE) for closure of Ti@C_{2n} from collision of **I2** and He as well as for the shrinkage of Ti@C_{28} systems depending on the initial structures. The KE needed for the closure of the cage when initial structures are taken from Car-Parrinello MD simulations at 2000 K (distorted structures) are significantly smaller than those needed to close the cage when optimized **I2** structures at 0 K were used. No significant differences in KE are found for cage shrinkage when starting with optimized or distorted cages.

Event	Initial structure	KE He* (eV)	KE transf** (eV)	Extra KE b) (eV)
**I2 to Ti@C_{28}**	Optimized 0K	14.2	11.9	7.4
	Dynamics 2000K	11.2	9.1	4.6
**Ti@C_{30} to I1'**	Optimized 0K	32.8	25.5	19.6
	Dynamics 2000K	31.9	25.0	19.1
**I1' to Ti@C_{28}**	Optimized 0K	20.6	15.9	13.0
	Dynamics 2000K	17.2	13.4	10.5
**I2 to Ti@C_{48}**	Optimized 0K	9.1	6.5	4.4
	Dynamics 2000K	8.5	6.3	4.2

* Kinetic energies (KE in eV) for the He atom required to close the cage. ** Kinetic energy (in eV) transferred from the He atom to the carbon cluster. a) Gibbs free energy barrier (in eV) for each system. b) Extra kinetic energy, KE transferred - $\Delta G^{\ddagger}$, (in eV) mainly dissipated as vibrational energy.

Movie1 Collision with He atom to Ti@C_{30} cage using Car-Parrinello Molecular Dynamics. A loss of a carbon atom forming the intermediate **I1'** is observed during the trajectory. In this case, He velocity is 39700 $m{\cdot}s^{-1}$ (kinetic energy around 33 eV).

Movie2 Collision with He atom to intermediate **I2** to form Ti@C_{28}. The velocity of the He atom required to close the cage is 27600 $m{\cdot}s^{-1}$ (kinetic energy around 16 eV) in this Car-Parrinello MD.

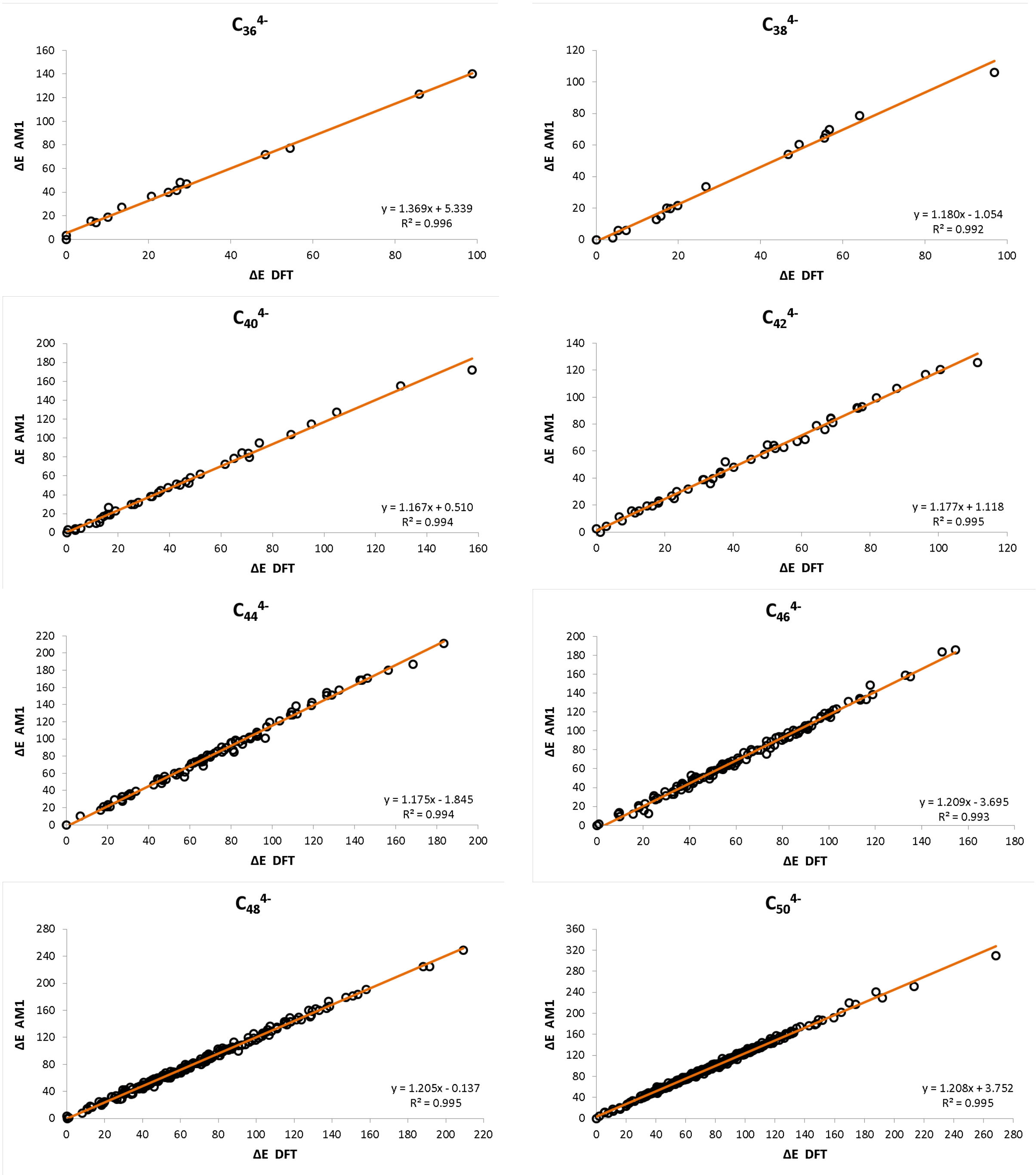

Fig. S11 AM1 vs DFT (BP86/TZP) relative energies (in kcal mol^{-1}) for the C_{2n}^{4-} (2n=36-50). Very good linear correlations between the AM1 and DFT energies are found for almost all of the families, with slopes rather near to 1 (around 1.15-1.20). Only in the case of 2n=36, the slope is somewhat larger (1.37). This means that in this least favorable case, a relative energy of 20 kcal·mol^{-1} at AM1 level corresponds to 15 kcal·mol^{-1} at DFT level.

Strategy to find out the lowest-energy endohedral metallofullerenes, Ti@C_{2n} in the present case, for larger cages (2n > 50).

1) Compute all the isomers at AM1 level.
2) Re-optimize at BP86/TZP level those tetraanions in a range of 30 kcal·mol^{-1} with respect to the most stable isomer.
3) Carry out at BP86/TZP level an accurate exploration of the different possible positions of the metal atom or cluster (Ti in this case) inside the cage.

xyz coordinates. For other structures that not appear here, please contact the authors.

(i) xyz coordinates of the optimized structures in Ti@C_{26} + C_2 to Ti@C_{28} profile (Figure 7).

1) Reactants (R)

C_2

C	0.000000	0.000000	-0.656185
C	0.000000	0.000000	0.656185

Ti@D_{3h}-C_{26}(1)

Ti	-1.258784	0.391725	-1.056840
C	0.274499	1.300669	0.080493
C	-0.132273	-0.032976	0.617471
C	-1.565179	0.000147	0.920976
C	-2.089072	1.326174	0.583357
C	-0.969010	2.164316	0.058890
C	0.936660	1.060059	-1.188427
C	0.835487	-0.441800	-1.502717
C	0.114040	-1.082397	-0.417783
C	-1.229596	-1.697639	-0.748918
C	-2.246757	-1.001406	0.096572
C	-3.233852	-0.305424	-0.783716
C	-3.133566	1.173361	-0.474557
C	-2.759572	2.055586	-1.565595
C	-1.395882	2.680094	-1.229354
C	-0.601034	2.613053	-2.422324
C	0.624326	1.761363	-2.400727
C	0.495619	0.779605	-3.467224
C	0.468305	-0.553851	-2.885360
C	-0.855811	-1.160779	-3.212097
C	-1.684547	-1.596003	-2.124077
C	-2.946825	-0.718999	-2.146087
C	-2.802518	0.191707	-3.245886
C	-2.704097	1.649850	-2.940859
C	-1.450602	2.131583	-3.501389
C	-0.786727	0.995284	-4.083364
C	-1.606828	-0.183140	-3.985352

2) Intermediates (I1, I2)

I1

Ti	0.093014	0.380582	0.843060
C	0.930624	-0.616723	2.495377
C	1.181045	-1.328241	1.207955
C	-0.116158	-1.650842	0.605477
C	-1.176339	-1.194653	1.480836
C	-0.568090	-0.521289	2.645941
C	1.681998	0.633573	2.436666
C	2.277504	0.749812	1.048578
C	1.935412	-0.434331	0.276299
C	1.045632	-0.215179	-0.933572
C	-0.211011	-0.977821	-0.693614
C	-1.365243	-0.053216	-0.679168
C	-2.214980	-0.267698	0.702764
C	-2.046438	1.037718	1.555651
C	-1.142215	0.812459	2.731289
C	-0.361574	2.009579	2.915200
C	1.109489	1.922072	2.746984
C	1.490967	2.864532	1.703625
C	2.081243	2.122607	0.595395
C	1.201804	2.313413	-0.577750
C	0.641365	1.154685	-1.222070
C	-0.876738	1.245780	-1.042177
C	-1.154349	2.470538	-0.327433
C	-1.747075	2.336212	1.035187
C	-0.849425	3.002306	1.969581
C	0.283037	3.475362	1.220494
C	0.101550	3.187408	-0.176607
C	-4.815569	-0.865666	0.714108
C	-3.584122	-0.714971	0.531535

I2

Ti	-1.511245	0.465605	-1.149994
C	0.047344	1.362253	0.136867
C	-0.532132	0.111451	0.682325
C	-2.001098	0.215131	0.788664
C	-2.327325	1.624690	0.358094
C	-1.078054	2.287665	-0.087245
C	0.867284	1.022108	-0.999410
C	0.737547	-0.462733	-1.226032
C	-0.151984	-0.995161	-0.223631
C	-1.409510	-1.613749	-0.680176
C	-2.553388	-0.932013	-0.026010
C	-3.601425	-0.690900	-1.078129
C	-3.311000	1.792061	-0.741106
C	-2.614151	2.301117	-1.901345
C	-1.263157	2.782669	-1.444584
C	-0.343718	2.557539	-2.509997
C	0.811605	1.662620	-2.282435
C	0.767836	0.614374	-3.293280
C	0.601718	-0.671031	-2.638453
C	-0.680614	-1.253883	-3.087252
C	-1.648006	-1.630772	-2.113799
C	-2.903565	-0.833950	-2.361078
C	-2.579785	0.135846	-3.416069
C	-2.439597	1.658530	-3.198921
C	-1.108715	2.026479	-3.642959
C	-0.434466	0.834869	-4.060176
C	-1.316672	-0.295298	-3.985385
C	-5.040152	-0.227903	-0.809533
C	-4.358994	0.858209	-0.829641

3) Transition states (TS1, TS2)

TS1

Ti	0.119789	0.525764	0.014102
C	0.431372	1.259462	-1.964524
C	0.554835	2.309910	-0.914421
C	-0.714606	2.390945	-0.186533
C	-1.648313	1.421437	-0.757031
C	-0.964781	0.683896	-1.833695
C	1.541704	0.340188	-1.782946
C	2.306303	0.757339	-0.547303
C	1.635082	1.930891	0.040230
C	0.989348	1.776265	1.397705
C	-0.474554	2.061180	1.221123
C	-1.285739	0.874527	1.598245
C	-2.188675	0.459891	0.350247
C	-1.793044	-0.969181	-0.273600
C	-1.098346	-0.735217	-1.609406
C	0.014320	-1.642560	-1.635006
C	1.390677	-1.097543	-1.762358
C	2.118822	-1.604160	-0.623836
C	2.532458	-0.459141	0.213434
C	1.944706	-0.612751	1.537815
C	1.065930	0.454583	2.013196
C	-0.337938	-0.106000	2.111995
C	-0.267637	-1.479929	1.659532
C	-1.013266	-1.946936	0.466443
C	-0.036095	-2.518124	-0.465337
C	1.249882	-2.448173	0.152295
C	1.117081	-1.829709	1.455687
C	-4.017446	-1.224925	-0.221285
C	-3.530720	-0.146940	0.302985

TS2

Ti	-0.265673	0.212540	0.321512
C	0.101887	2.392773	0.349913
C	-0.499665	1.884184	1.591643
C	-1.768433	1.171394	1.296889
C	-1.980470	1.283454	-0.166387
C	-0.822468	2.043401	-0.739051
C	1.460591	1.897089	0.253799
C	1.677344	0.974191	1.409798
C	0.472367	0.944950	2.210454
C	-0.209591	-0.337971	2.390331
C	-1.626051	-0.209327	1.842117
C	-1.826872	-1.330869	0.911616
C	-2.152079	0.068652	-1.003047
C	-1.092689	0.050981	-1.956807
C	-0.295764	1.297269	-1.848746
C	1.073195	0.917477	-1.996310
C	1.999912	1.243541	-0.912086
C	2.640782	0.012102	-0.510391
C	2.346014	-0.207031	0.887396
C	1.660869	-1.477085	1.046890
C	0.422170	-1.504626	1.771800
C	-0.579159	-2.042120	0.837888
C	0.002628	-2.173115	-0.473712
C	-0.256625	-1.092498	-2.204589
C	1.106106	-0.530600	-2.266082
C	1.997349	-1.055558	-1.279471
C	1.400323	-1.992211	-0.355974
C	-2.016878	-2.427833	-1.116824
C	-2.427355	-1.268732	-0.455170

4) Product (P)

Ti@T_d-C_{28}(2)

Ti	0.463057	-0.192335	-0.003878
C	-0.539452	-1.036506	2.115382
C	-0.784873	0.364060	2.385585
C	0.430268	1.102797	2.120122
C	1.510494	0.147320	1.872010
C	0.890582	-1.218257	1.869729
C	-1.517982	-1.489944	1.153132
C	-2.325650	-0.310346	0.735517
C	-1.812420	0.831462	1.452294
C	-1.290249	1.971284	0.739268
C	0.128285	2.139946	1.159487
C	0.933335	2.246333	-0.036510
C	2.016901	1.297585	-0.296055
C	2.364742	0.266715	0.686687
C	2.330895	-1.044951	-0.017220
C	1.363755	-1.934862	0.682462
C	0.360270	-2.348787	-0.301407
C	-1.067077	-2.162266	-0.043997
C	-1.657091	-1.498567	-1.186649
C	-2.339308	-0.293956	-0.706642
C	-1.820176	0.839273	-1.432320
C	-1.309978	1.974182	-0.702981
C	0.046363	2.254814	-1.179758
C	0.436819	1.184476	-2.072206
C	1.699892	0.619855	-1.595998
C	1.852802	-0.826105	-1.409777
C	0.662855	-1.661411	-1.598489
C	-0.593795	-1.084921	-2.076424
C	-0.714176	0.338661	-2.295177

(ii) xyz coordinates of the optimized structures of the Ti@C_{2n} cages that appear in Fig. 9.

1) Ti@$\boldsymbol{D_{3h}}$-C_{26}(1)

Ti	-1.258784	0.391725	-1.056840
C	0.274499	1.300669	0.080493
C	-0.132273	-0.032976	0.617471
C	-1.565179	0.000147	0.920976
C	-2.089072	1.326174	0.583357
C	-0.969010	2.164316	0.058890
C	0.936660	1.060059	-1.188427
C	0.835487	-0.441800	-1.502717
C	0.114040	-1.082397	-0.417783
C	-1.229596	-1.697639	-0.748918
C	-2.246757	-1.001406	0.096572
C	-3.233852	-0.305424	-0.783716
C	-3.133566	1.173361	-0.474557
C	-2.759572	2.055586	-1.565595
C	-1.395882	2.680094	-1.229354
C	-0.601034	2.613053	-2.422324
C	0.624326	1.761363	-2.400727
C	0.495619	0.779605	-3.467224
C	0.468305	-0.553851	-2.885360
C	-0.855811	-1.160779	-3.212097
C	-1.684547	-1.596003	-2.124077
C	-2.946825	-0.718999	-2.146087
C	-2.802518	0.191707	-3.245886
C	-2.704097	1.649850	-2.940859
C	-1.450602	2.131583	-3.501389
C	-0.786727	0.995284	-4.083364
C	-1.606828	-0.183140	-3.985352

2) Ti@$\boldsymbol{T_d}$-C_{28}(2)

Ti	0.463057	-0.192335	-0.003878
C	-0.539452	-1.036506	2.115382
C	-0.784873	0.364060	2.385585
C	0.430268	1.102797	2.120122
C	1.510494	0.147320	1.872010
C	0.890582	-1.218257	1.869729
C	-1.517982	-1.489944	1.153132
C	-2.325650	-0.310346	0.735517
C	-1.812420	0.831462	1.452294
C	-1.290249	1.971284	0.739268
C	0.128285	2.139946	1.159487
C	0.933335	2.246333	-0.036510
C	2.016901	1.297585	-0.296055
C	2.364742	0.266715	0.686687
C	2.330895	-1.044951	-0.017220
C	1.363755	-1.934862	0.682462
C	0.360270	-2.348787	-0.301407
C	-1.067077	-2.162266	-0.043997
C	-1.657091	-1.498567	-1.186649
C	-2.339308	-0.293956	-0.706642
C	-1.820176	0.839273	-1.432320
C	-1.309978	1.974182	-0.702981
C	0.046363	2.254814	-1.179758
C	0.436819	1.184476	-2.072206
C	1.699892	0.619855	-1.595998
C	1.852802	-0.826105	-1.409777
C	0.662855	-1.661411	-1.598489
C	-0.593795	-1.084921	-2.076424
C	-0.714176	0.338661	-2.295177

3) Ti@$\boldsymbol{C_{2v}}$-C_{30}(3)

Ti	-0.254921	0.142966	-0.171491
C	2.827555	0.088096	-0.753860
C	1.926009	-2.011179	0.256746
C	1.289831	-2.162897	1.553035
C	-0.107096	-2.375580	1.341035
C	-0.842203	-1.251949	1.875054
C	-1.866157	-0.862958	0.908756
C	-2.141998	0.525462	0.584324
C	-2.120889	0.620536	-0.902146
C	-1.178855	1.689960	-1.302734
C	-0.173848	1.065717	-2.170145
C	1.217170	1.293844	-1.944720
C	1.879930	-0.025569	-1.818518
C	2.760087	1.438215	-0.205328
C	1.703774	2.148123	-0.876592
C	0.718097	2.509857	0.125679
C	-0.681117	2.301045	-0.065535
C	-1.223975	1.538656	1.111319
C	-0.047955	1.155031	1.950980
C	0.139663	-0.246276	2.333544
C	1.458284	-0.872362	2.197116
C	2.361161	1.224023	1.181232
C	1.103152	1.866096	1.404144
C	0.889881	-2.073125	-0.778563
C	-0.371445	-2.399903	-0.079711
C	-1.563478	-1.598766	-0.345261
C	-1.648235	-0.676390	-1.463210
C	-0.421235	-0.416388	-2.219588
C	0.868816	-1.077102	-1.852046
C	2.522650	-0.159316	1.537668
C	2.819351	-0.881543	0.307070

4) Ti@$\boldsymbol{D_3}$-C_{32}(6)

Ti	-0.000084	0.000070	-0.807157
C	-2.301667	-0.648431	0.440018
C	-2.316887	0.658792	-0.199273
C	-1.714827	1.769950	0.530029
C	-0.684682	2.378195	-0.315856
C	0.589337	2.317674	0.439886
C	1.729086	1.677230	-0.199402
C	2.390361	0.600175	0.529969
C	2.402027	-0.596108	-0.315820
C	1.712496	-1.669155	0.439924
C	0.587992	-2.335909	-0.199279
C	-0.675359	-2.370079	0.530098
C	-1.717120	-1.782046	-0.315733
C	-1.836262	-0.774114	1.798861
C	-0.807027	-1.827326	1.835942
C	0.337402	-1.299909	2.523387
C	1.588265	-1.203174	1.798756
C	1.985714	0.214754	1.835723
C	0.956837	0.942102	2.523253
C	0.247710	1.977115	1.798740
C	-1.178994	1.612397	1.835845
C	-1.294364	0.357550	2.523394
C	-0.000037	-0.000046	3.069623
C	-1.852767	0.777919	-1.603031
C	-0.809914	1.839811	-1.636827
C	0.353848	1.350962	-2.364873
C	1.600119	1.215618	-1.603161
C	1.998267	-0.218432	-1.636842
C	0.993055	-0.981892	-2.364850
C	0.252724	-1.993492	-1.603009
C	-1.188319	-1.621344	-1.636659
C	-1.346752	-0.369061	-2.364806
C	0.000073	-0.000005	-2.865752

5) Ti@C_2-C_{34}(5)

Ti	-0.198391	-1.064448	-0.138596
C	-0.419046	-3.070111	0.066556
C	-0.999187	-2.634894	-1.236810
C	-2.094542	-1.689504	-1.001574
C	-2.285712	-1.637405	0.431809
C	-1.215100	-2.392639	1.118965
C	1.008201	-2.749069	-0.009404
C	1.268341	-2.077049	-1.316523
C	0.015470	-1.960654	-2.049562
C	-0.483304	-0.603928	-2.351863
C	-1.834313	-0.443827	-1.728711
C	-2.177958	0.832400	-1.102304
C	-2.514352	0.832257	0.305212
C	-2.446705	-0.361735	1.060615
C	-1.413293	-0.209869	2.077613
C	-0.592378	-1.395395	2.084822
C	0.848786	-1.191025	2.041274
C	1.642439	-1.928482	1.007170
C	2.449293	-0.911071	0.341210
C	2.101685	-0.896236	-1.066869
C	1.748661	0.363247	-1.681816
C	0.438062	0.505474	-2.315034
C	-0.060184	1.838981	-1.960471
C	-1.316639	1.961895	-1.308294
C	-1.049056	2.644397	0.003031
C	-1.674429	1.851568	0.985562
C	-0.910449	1.172042	2.017451
C	0.506525	1.358807	2.038840
C	1.366958	0.146435	2.114196
C	2.394461	0.300835	1.100493
C	2.231877	1.551029	0.462343
C	2.006937	1.618645	-0.955234
C	0.948159	2.546914	-1.212021
C	0.348095	2.902790	0.070943
C	1.118060	2.273967	1.109175

6) Ti@D_{2d}-C_{36}(14)

Ti	0.568785	0.581702	0.326105
C	-0.804309	2.648366	0.019178
C	0.657225	2.701523	-0.096935
C	1.235867	2.303466	1.189877
C	0.104165	1.798219	2.011499
C	-1.165020	2.012706	1.273036
C	-1.368924	2.227381	-1.222669
C	-0.256981	1.766720	-2.050034
C	0.981970	2.044944	-1.393187
C	2.034162	1.009299	-1.388036
C	2.689343	0.701523	-0.087244
C	2.275942	1.279933	1.194647
C	1.784997	0.144092	2.019374
C	0.439768	0.460450	2.505478
C	-0.577282	-0.573433	2.423455
C	-1.920079	-0.245170	1.967276
C	-2.181980	1.003432	1.273107
C	-2.824709	0.663925	0.006773
C	-2.356591	1.204581	-1.201388
C	-1.893591	0.076626	-2.056118
C	-0.600551	0.404353	-2.538153
C	0.425069	-0.604889	-2.533154
C	1.779511	-0.237080	-2.040408
C	2.253861	-1.337344	-1.205313
C	2.659543	-0.760131	0.035714
C	2.023670	-1.124800	1.288227
C	1.030794	-2.157846	1.288442
C	-0.225219	-1.912675	1.975435
C	-1.311050	-2.358093	1.114346
C	-2.343679	-1.341947	1.109369
C	-2.799656	-0.815085	-0.112680
C	-2.159437	-1.179140	-1.364356
C	-1.139078	-2.183238	-1.359385
C	0.115794	-1.900576	-2.046316
C	1.246835	-2.341161	-1.183935
C	0.707914	-2.811940	0.023885
C	-0.770988	-2.811359	-0.102849

7) Ti@D_{6h}-C_{36}(15)

Ti	0.014684	0.088723	1.007311
C	-1.165182	-2.030446	-1.474363
C	-0.716200	-1.264589	-2.653401
C	-1.436941	-0.028086	-2.678793
C	-2.322389	-0.004682	-1.524839
C	-2.169043	-1.250866	-0.785467
C	-0.005687	-2.544328	-0.760531
C	1.151583	-2.041404	-1.485722
C	0.698289	-1.271226	-2.660326
C	1.430356	-0.041538	-2.692682
C	0.710878	1.198017	-2.703239
C	-0.705950	1.204693	-2.696405
C	-1.147676	2.011633	-1.546999
C	-2.158397	1.266424	-0.830231
C	-2.150633	1.293219	0.595071
C	-2.308666	0.050437	1.342464
C	-2.157929	-1.226599	0.639068
C	-1.154160	-1.986145	1.363527
C	0.001157	-2.540859	0.657452
C	1.168467	-1.996891	1.352294
C	2.172058	-1.246639	0.618107
C	2.169152	-1.271104	-0.806468
C	2.327090	-0.026442	-1.547306
C	2.181778	1.246152	-0.851163
C	1.171257	2.000777	-1.558206
C	0.017658	2.539277	-0.851414
C	0.024742	2.586167	0.565878
C	-1.135803	2.072341	1.295918
C	-0.706373	1.348618	2.500082
C	-1.452062	0.069383	2.523331
C	-0.713502	-1.212432	2.527704
C	0.746398	-1.219173	2.520561
C	1.496705	0.055721	2.508885
C	2.341767	0.028879	1.320028
C	2.188034	1.273012	0.574108
C	1.187364	2.061574	1.284706
C	0.762755	1.341742	2.492851

8) Ti@C_2-C_{38}(17)

Ti	0.882243	0.048624	0.511859
C	1.974849	1.844121	-0.886027
C	2.789677	0.725269	-0.440947
C	2.772932	0.691348	1.009396
C	1.790805	1.713444	1.453479
C	1.258500	2.381073	0.244819
C	1.313734	1.500021	-2.094765
C	1.663711	0.118525	-2.415086
C	2.456829	-0.415801	-1.340881
C	1.991696	-1.616342	-0.687673
C	2.074070	-1.698830	0.792153
C	2.440651	-0.568190	1.666446
C	1.276127	-0.314069	2.546941
C	0.868881	1.112307	2.430384
C	-0.529285	1.376008	2.271262
C	-1.010561	2.237200	1.189749
C	-0.160576	2.575391	0.093882
C	-0.867553	2.189962	-1.151880
C	-0.122135	1.666258	-2.250044
C	-0.610598	0.464351	-2.900910
C	0.482700	-0.526012	-2.882123
C	-0.014722	-1.716905	-2.258047
C	0.697628	-2.203382	-1.101465
C	-0.046519	-2.561314	0.108543
C	0.778575	-2.192749	1.238908
C	0.267879	-1.352931	2.308395
C	-1.135860	-1.056453	2.318390
C	-1.532489	0.335143	2.429829
C	-2.609615	0.569539	1.479669
C	-2.263245	1.693078	0.673847
C	-2.165452	1.594304	-0.758869
C	-2.595020	0.376186	-1.395615
C	-1.784962	-0.162395	-2.463302
C	-1.453402	-1.564757	-2.135078
C	-2.156457	-1.904545	-0.972194
C	-1.467391	-2.400070	0.201183
C	-1.995370	-1.684805	1.344895
C	-2.931626	-0.668778	0.846491
C	-2.971855	-0.747328	-0.557866

9) Ti@D_2-C_{40}(38)

Ti	0.526521	-0.666742	-0.816232
C	0.362014	0.562485	-3.001800
C	-0.347975	-0.716695	-2.995678
C	0.554243	-1.757843	-2.593065
C	1.869758	-1.118545	-2.295788
C	1.680153	0.345217	-2.409966
C	-0.525125	1.579329	-2.590269
C	-1.814175	0.941697	-2.279051
C	-1.656108	-0.487664	-2.405532
C	-2.036826	-1.328770	-1.311022
C	-1.136298	-2.432328	-0.867725
C	0.171427	-2.639472	-1.479113
C	1.317887	-2.618117	-0.540655
C	2.335799	-1.663849	-1.023981
C	2.634276	-0.763291	0.087027
C	2.693824	0.679052	-0.092501
C	2.043540	1.222493	-1.327281
C	1.155459	2.308556	-0.918524
C	-0.163233	2.450528	-1.490107
C	-1.283986	2.495291	-0.589991
C	-2.251540	1.488677	-1.051045
C	-2.642078	0.667618	0.059179
C	-2.650902	-0.761133	-0.119080
C	-2.270544	-1.597494	0.996174
C	-1.323018	-2.601702	0.550780
C	-0.187981	-2.547216	1.462931
C	1.112752	-2.392688	0.877601
C	2.003292	-1.303546	1.305470
C	1.659785	-0.458053	2.399816
C	1.821823	0.960537	2.214706
C	2.303045	1.502562	0.992940
C	1.305604	2.462827	0.499164
C	0.176547	2.457687	1.394114
C	-1.130457	2.338195	0.817561
C	-2.036123	1.244050	1.262409
C	-1.655037	0.406786	2.340036
C	-1.814470	-1.024408	2.200328
C	-0.535252	-1.660794	2.543459
C	0.348819	-0.663830	2.991995
C	-0.359638	0.623441	2.966483
C	0.528430	1.602536	2.504177

10) Ti@D_3-C_{42}(45)

Ti	0.000000	0.000000	-1.280552
C	-0.668953	2.495851	1.831144
C	0.655172	2.849759	1.256963
C	1.587313	1.933468	1.793985
C	0.923192	1.063852	2.729746
C	-0.500744	1.352542	2.674387
C	-1.609029	2.506535	0.790746
C	-0.938613	2.901884	-0.438736
C	0.502612	3.032441	-0.139256
C	1.398803	2.325524	-0.997175
C	2.344768	1.337620	-0.453716
C	2.422289	1.104736	0.937668
C	2.405942	-0.255775	1.453117
C	1.423723	-0.268371	2.543530
C	0.500744	-1.352542	2.674387
C	-0.923192	-1.063852	2.729746
C	-1.423723	0.268371	2.543530
C	-2.405942	0.255775	1.453117
C	-2.445555	1.356544	0.530942
C	-2.386446	1.119856	-0.888210
C	-1.413960	2.096598	-1.495988
C	-0.510817	1.666117	-2.550482
C	0.923559	1.811303	-2.252874
C	1.618085	0.570691	-2.580019
C	2.396310	0.208557	-1.401328
C	2.386446	-1.119856	-0.888210
C	2.445555	-1.356544	0.530942
C	1.609029	-2.506535	0.790746
C	0.668953	-2.495851	1.831144
C	-0.655172	-2.849759	1.256963
C	-1.587313	-1.933468	1.793985
C	-2.422289	-1.104736	0.937668
C	-2.344768	-1.337620	-0.453716
C	-2.396310	-0.208557	-1.401328
C	-1.618085	-0.570691	-2.580019
C	-0.660344	0.343721	-3.183515
C	0.660344	-0.343721	-3.183515
C	0.510817	-1.666117	-2.550482
C	1.413960	-2.096598	-1.495988
C	0.938613	-2.901884	-0.438736
C	-0.502612	-3.032441	-0.139256
C	-1.398803	-2.325524	-0.997175
C	-0.923559	-1.811303	-2.252874

11) Ti@C_1-C_{42}(33)

Ti	-0.207544	-0.389198	0.938325
C	2.814397	1.102791	-1.453674
C	3.095890	-0.301113	-1.650768
C	3.215785	-0.916205	-0.366202
C	3.207567	0.144991	0.626967
C	2.944312	1.409851	-0.076425
C	1.587313	1.431493	-2.142648
C	1.031271	0.179664	-2.686390
C	1.959026	-0.860982	-2.315658
C	1.470809	-1.974066	-1.536984
C	2.243433	-1.974506	-0.273875
C	1.512222	-2.137268	0.931540
C	1.635747	-1.141093	2.016888
C	2.376735	0.079229	1.755603
C	1.581533	1.343912	1.799623
C	1.902491	2.114645	0.611800
C	0.833364	2.707439	-0.131862
C	0.700918	2.365052	-1.570583
C	-0.683467	2.282687	-1.881149
C	-1.267970	1.075796	-2.449462
C	-0.391544	-0.067674	-2.711387
C	-0.845848	-1.381307	-2.277626
C	0.094971	-2.229579	-1.539184
C	-0.586485	-2.672353	-0.318344
C	0.127206	-2.615205	0.932475
C	-0.554166	-2.058369	2.108096
C	0.390951	-1.135766	2.766373
C	-0.297892	0.138821	2.953847
C	0.261561	1.347815	2.335183
C	-0.814983	2.017877	1.586690
C	-0.511462	2.746666	0.395218
C	-1.442172	2.630135	-0.708141
C	-2.573567	1.783388	-0.645118
C	-2.536356	0.880644	-1.808381
C	-2.931696	-0.397569	-1.361624
C	-2.131218	-1.543503	-1.639875
C	-1.963039	-2.285856	-0.413080
C	-2.545649	-1.484537	0.663833
C	-1.846583	-1.362822	1.922772
C	-1.664522	-0.001876	2.460488
C	-2.003720	1.157158	1.652576
C	-2.869912	1.007296	0.516848
C	-3.189034	-0.346696	0.073643

12) Ti@D_2-C_{44}(89)

Ti	0.760102	0.469107	-0.902947
C	1.796026	-0.442773	-2.551149
C	2.680983	0.400077	-1.746772
C	2.137559	1.758665	-1.727983
C	0.906713	1.744526	-2.539430
C	0.695587	0.374949	-3.063172
C	1.520489	-1.669089	-1.801513
C	2.283525	-1.615501	-0.561937
C	2.939726	-0.297082	-0.498188
C	2.981104	0.400124	0.752806
C	2.280345	1.725839	0.792626
C	1.807698	2.330364	-0.419234
C	0.402587	2.772541	-0.454639
C	-0.164751	2.364909	-1.744614
C	-1.481415	1.796065	-1.772476
C	-1.749741	0.620995	-2.564994
C	-0.614902	-0.188448	-3.020956
C	-0.823333	-1.542726	-2.548976
C	0.209989	-2.229362	-1.799942
C	-0.364114	-2.681715	-0.537659
C	0.403676	-2.706876	0.642799
C	1.800982	-2.229682	0.622556
C	2.069116	-1.602315	1.873207
C	2.659734	-0.281217	1.940166
C	1.776618	0.545706	2.739232
C	1.508909	1.755950	2.011004
C	0.175710	2.295890	1.990024
C	-0.382801	2.761217	0.722762
C	-1.772682	2.289184	0.655696
C	-2.271365	1.730644	-0.546361
C	-2.938345	0.421941	-0.538899
C	-2.587900	-0.231146	-1.779190
C	-2.022104	-1.543709	-1.760049
C	-1.759137	-2.206630	-0.505029
C	-2.261780	-1.653380	0.684726
C	-1.475052	-1.727301	1.914647
C	-0.172350	-2.280173	1.904677
C	0.870571	-1.606712	2.663777
C	0.674722	-0.277180	3.180077
C	-0.640098	0.280184	3.177307
C	-0.853066	1.639616	2.724003
C	-2.055317	1.641660	1.896534
C	-2.589343	0.329633	1.881173
C	-2.930390	-0.328408	0.658864
C	-1.713823	-0.517980	2.662368

13) Ti@C_1-C_{46}(114)

Ti	-0.104513	0.577381	0.618081
C	-0.540983	2.629483	0.891475
C	-0.871424	1.820867	2.085543
C	-1.978059	0.966122	1.714798
C	-2.455697	1.360622	0.409223
C	-1.529527	2.336464	-0.142037
C	0.885586	2.500988	0.579719
C	1.421752	1.657102	1.665256
C	0.336929	1.177570	2.546450
C	0.421261	-0.283861	2.584449
C	-0.736253	-1.175570	2.273810
C	-1.928611	-0.494760	1.754097
C	-2.575130	-0.963362	0.528873
C	-2.837470	0.185821	-0.329074
C	-2.538344	0.133041	-1.708710
C	-1.815188	1.228414	-2.317882
C	-1.135739	2.198351	-1.491200
C	0.287371	2.202815	-1.853519
C	1.288688	2.197408	-0.793855
C	2.475279	1.349329	-0.981663
C	2.947267	0.477384	0.118103
C	2.279316	0.550013	1.355154
C	1.721434	-0.637234	1.987742
C	1.965829	-1.915571	1.431824
C	0.934175	-2.885801	1.469170
C	-0.442524	-2.466719	1.737862
C	-1.248353	-2.996331	0.657811
C	-2.190055	-2.198440	-0.048482
C	-1.894728	-2.268277	-1.480226
C	-2.024880	-1.112111	-2.281832
C	-0.997930	-0.758627	-3.246038
C	-0.867480	0.694952	-3.239694
C	0.410056	1.296819	-2.989433
C	1.580208	0.509556	-3.149265
C	2.657433	0.644177	-2.188103
C	3.141712	-0.697422	-1.869397
C	3.218487	-0.833040	-0.443570
C	2.711610	-2.010044	0.168344
C	2.013988	-3.002620	-0.636571
C	0.952667	-3.546592	0.183547
C	-0.361140	-3.607009	-0.306116
C	-0.703315	-3.104261	-1.619395
C	0.340197	-2.710935	-2.475387
C	0.170744	-1.536282	-3.339386
C	1.443034	-0.878410	-3.451199
C	2.394078	-1.616411	-2.625863
C	1.737767	-2.724829	-2.001984

14) Ti@C_1-C_{48}(196)

Ti	-0.438437	0.374771	-1.374668
C	2.594186	-2.083941	-0.682349
C	1.667945	-3.011624	-0.044610
C	1.645477	-2.724495	1.342511
C	2.563268	-1.636325	1.605033
C	3.107878	-1.200020	0.353697
C	1.970154	-1.547804	-1.827433
C	0.673061	-2.230654	-1.983430
C	0.448420	-3.017670	-0.777708
C	-0.796933	-2.901921	-0.097292
C	-0.816553	-2.626875	1.334366
C	0.412087	-2.456893	2.019451
C	0.616611	-1.245111	2.824820
C	1.949045	-0.720445	2.538824
C	2.106840	0.658323	2.317443
C	2.856002	1.097557	1.155118
C	3.193026	0.191806	0.105402
C	2.720912	0.753299	-1.172892
C	2.049269	-0.113304	-2.126155
C	1.007447	0.439290	-2.987267
C	-0.193021	-0.350924	-3.328639
C	-0.407431	-1.597256	-2.623952
C	-1.716215	-1.537523	-1.969237
C	-1.891252	-2.161729	-0.693179
C	-2.629054	-1.481789	0.343676
C	-1.935315	-1.738571	1.607063
C	-1.781965	-0.685305	2.534164
C	-0.453777	-0.378442	3.063765
C	-0.257363	1.071606	2.956170
C	0.971159	1.555422	2.469963
C	0.965623	2.489212	1.343416
C	2.148322	2.199870	0.576729
C	2.115560	2.032744	-0.839329
C	0.932577	2.446030	-1.499588
C	0.512363	1.779989	-2.706176
C	-0.944569	1.823823	-2.790745
C	-1.410234	0.485504	-3.179802
C	-2.315117	-0.224521	-2.266724
C	-2.946173	0.467379	-1.189207
C	-3.189108	-0.194425	0.078093
C	-3.140814	0.811027	1.098928
C	-2.432505	0.574918	2.283829
C	-1.474502	1.648105	2.481272
C	-1.513975	2.499827	1.335299
C	-0.271696	2.849060	0.700773
C	-0.253480	2.840872	-0.758863
C	-1.412136	2.418432	-1.546539
C	-2.561348	1.889867	-0.860044
C	-2.663695	2.062188	0.521577
